# Atomically precise vanadium-oxide clusters

**DOI:** 10.1039/d0na00877j

**Published:** 2021-01-22

**Authors:** Sourav Chakraborty, Brittney E. Petel, Eric Schreiber, Ellen M. Matson

**Affiliations:** University of Rochester, Department of Chemistry Rochester NY 14627 USA ellen.matson@rochester.edu

## Abstract

Polyoxovanadate (POV) clusters are an important subclass of polyoxometalates with a broad range of molecular compositions and physicochemical properties. One relatively underdeveloped application of these polynuclear assemblies involves their use as atomically precise, homogenous molecular models for bulk metal oxides. Given the structural and electronic similarities of POVs and extended vanadium oxide materials, as well as the relative ease of modifying the homogenous congeners, investigation of the chemical and physical properties of pristine and modified cluster complexes presents a method toward understanding the influence of structural modifications (*e.g.* crystal structure/phase, chemical makeup of surface ligands, elemental dopants) on the properties of extended solids. This review summarises recent advances in the use of POV clusters as atomically precise models for bulk metal oxides, with particular focus on the assembly of vanadium oxide clusters and the consequences of altering the molecular composition of the assembly *via* organofunctionalization and the incorporation of elemental “dopants”.

## Abstract 2

Polyoxovanadate (POV) clusters are an important subclass of polyoxometalates with a broad range of molecular compositions and physicochemical properties.

## Graphical abstract

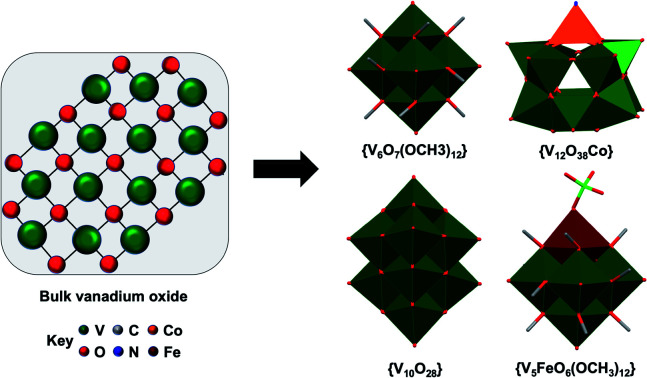

## Introduction

Vanadium oxides (*e.g.* V_2_O_5_, V_2_O_3_, VO_2_, *etc.*) represent an important subclass within reducible metal oxide (RMO) materials that are used in a wide variety of applications including electrical and optical devices, smart windows, and batteries, and as heterogenous catalysts for the activation of environmental contaminants and organic substrates.^1–6^ The widespread application of these materials is mainly credited to the range of oxidation states accessible by vanadium (*e.g.* V^II^ → V^V^) and its rich structural diversity, which has resulted in the isolation of an array of materials with distinct chemical properties. For example, of these vanadium-based materials, vanadium pentaoxide (V_2_O_5_) has attracted considerable interest in the field of heterogeneous catalysis due to its high activity in selective oxidation processes that result in the formation of alcohols, aldehydes, and ketones.^7–10^ In fact, Mars and Van Krevelen used this material to determine the oxidative Mars–Van Krevelen mechanism, which has provided a general foundation for understanding the role of metal–oxo moieties in substrate activation.^7^ In addition, extensive characterization of vanadium(iv) oxide (VO_2_) has revealed advantageous properties such as its reversible, temperature dependant metal-to-insulator phase transmission, which has resulted in the development of vanadium-based thermochromic devices.^4^

Given the widespread application of these materials, various analytical techniques have been used to analyse the structural, electronic, and optical properties of V_*x*_O_*y*_.^2,11–13^ For example, X-ray diffraction, X-ray photoelectron, Raman, atomic absorption, infrared, and electronic absorption spectroscopies, as well as transition electron or scanning electron microscopies, have all provided valuable insight into the surface structure or electronic properties of metal oxides pre- and post-catalysis. In addition, computational investigations are frequently used to gain atomistic information on the morphology and composition of bulk oxides.^14–16^ While these experimental and theoretical techniques, as well as the development of heterogeneous reaction and phase-change mechanisms, are continuously progressing, the level of understanding of the structural and electronic changes of extended solids, particularly *in situ*, remains limited. Spectroscopic resolution of bulk systems is complicated by thermal drift,^17,18^ artifact generation,^19,20^ and variations in surface properties due to oxygen–atom exchange and surface reconstruction.^21^ Thus, to gain a molecular-level understanding into the consequence of modifying bulk metal-oxides (*i.e.* alteration of surface ligands, addition of cationic or anionic dopants, *etc.*), researchers have sought to experimentally analyse multi-nuclear homogeneous complexes as atomically precise models of extended solids.

In this regard, polyoxometalates (POMs) stand out as intriguing models for metal oxide materials.^22–25^ These polynuclear assemblies are composed of multiple transition metal centres linked together through bridging oxygen atoms, with general formulas of [M_*x*_O_*y*_]^*n*−^ where M is commonly a high-valent, early-transition metal such as W^VI^, Mo^VI^, or V^V^. Like their bulk metal oxide congeners, these inorganic complexes can accommodate the addition or removal of reducing equivalents as a result of the redox-active nature of the metal ions that compose these clusters.^26^ Reduced derivatives of POMs stabilize reducing equivalents by delocalizing electron density across the assembly, resembling electronic communication between metals within the extended solid. However, due to their molecular nature, these clusters lack the band structure of semiconducting metal oxide materials. Despite this fact, given that both the atomic composition and physicochemical properties of POMs have been cited to resemble those of their nanocrystalline counterparts, structure–function investigations of these assemblies remain an important strategy for gaining information surrounding the consequences of modifying metal oxide materials.

**Fig. 1:**
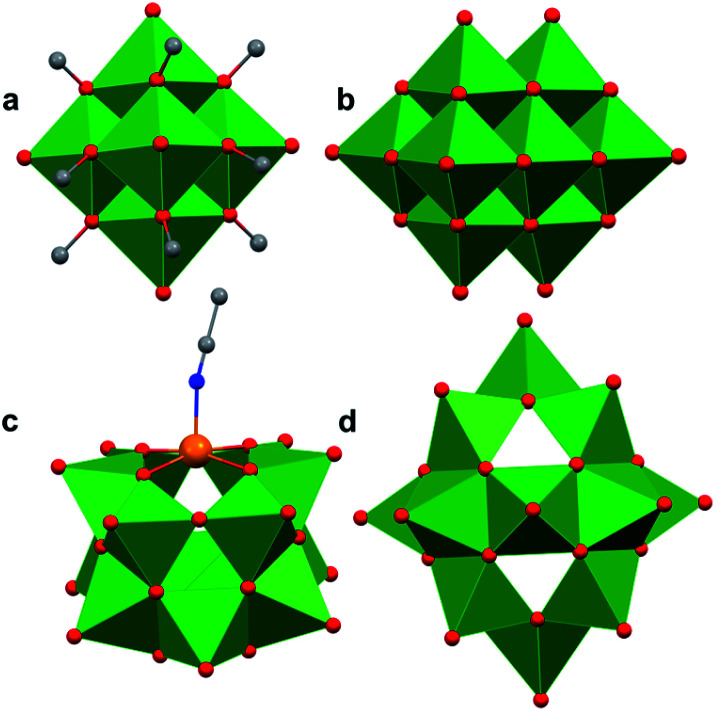
Popular structures of POV clusters: (a) [V_6_O_7_(OCH_3_)_12_]^1−^, (b) [V_10_O_28_]^6−^, (c) [V_12_O_38_Co]^2−^, and (d) [H_2_V_18_O_44_(N_3_)]^5−^. Colour code: V: green polyhedra, O: red, C: grey, N: blue, Co: orange. Hydrogen atoms, solvent molecules and counter cations are omitted for clarity.

Polyoxovanadates (POVs) represent a distinct subclass of POMs with versatile structures, redox profiles, and activities. In general, POVs can be divided into two sub-groups: (1) isopolyvanadates, which contain only vanadium and oxygen atoms [*e.g.* [V_10_O_28_]^6−^] and (2) heteropolyvanadates which contain heterometals (transition metals) along with vanadium and oxygen atoms in the structure [*e.g.* [V_5_O_6_(OCH_3_)_12_Fe(ClO_4_)], [V_12_O_38_Co]^2−^] (Fig. 1).^27,28^ H. T. Evans Jr was the first to report the structure of a POV, the plenary decavandate ion, [V_10_O_28_]^6−^, followed by M. T. Pope who reported the very first heteropolyvanadate [MV_13_O_38_]^7−^ (M = Mn^ii^, Ni^ii^) in the early 1970's.^29–34^ Since then, several derivatives of POVs have been reported, including [HV_4_O_12_]^3−^, [V_5_O_14_]^3−^, [H_3_V_10_O_28_]^3−^, [V_12_O_32_]^4−^, [V_13_O_34_]^3−^, [PV_14_O_42_]^9−^, [V_15_O_42_]^9−^, [HV_22_O_54_(ClO_4_)]^6−^, [H_2_V_18_O_44_(N_3_)]^5−^, [V_6_O_19−*x*_(OR)_*x*_]^*n*^, [V_34_O_82_]^10−^, *etc.*^28,35–50^ The coordination geometry of individual vanadium centres within a POV can vary from tetrahedral {VO_4_}, square-pyramidal {VO_5_}, to octahedral {VO_6_} geometries^51^ resulting in a wide range of structurally diverse metal oxide assemblies. Broadly speaking, the properties of POVs are influenced by the shape of the metal oxide cluster and the oxidation states of vanadium ions within the assembly (ranging from fully-oxidised (V^V^), mixed valent (V^V^/V^IV^ or V^IV^/V^III^), or fully reduced (V^IV^) oxidation state distributions). For an overview of synthetic routes to access an array of POV clusters in aqueous and non-aqueous media, their structural properties, and activity studies, the readers are encouraged to read articles by Y. Hayashi and P. Kögerler.^51,52^

While bulk vanadium oxides have shown high activities toward various heterogenous transformations, the modification or improvement of these catalysts toward the development and application of solid-state vanadium oxides requires an in-depth knowledge of the structural and electronic properties. Given the aforementioned limitations of current techniques, this review will summarise recent developments of molecular vanadium oxide cluster complexes as atomically precise models for vanadium oxide materials. For information regarding the application of POVs, the authors encourage readers to refer to a recent review article by Streb and co-workers describing the use of these clusters in energy storage and conversion.^53^ Our article will begin by describing details understood about the mechanisms of POV formation in aqueous and organic solvent. Specifically, we describe pathways through which vanadium oxide precursors can grow from mono- to multinuclear clusters depending on reaction conditions. Next, due to their prevalence in the POM/POV literature, the development and properties of organo-functionalised POVs will be described. These molecular systems provide an atomistic understanding of the role of organic ligands capping the surface of nanomaterials, such as particle dispersibility and charge transfer characteristics. We also describe an in-depth summary of the structural and electronic consequences of cluster modification *via* alteration of organic functionalities or through the introduction of cationic and anionic dopants. Through rigorous studies on the properties and reactivity of atomically precise, molecular POVs, a better understanding of structure–function relationships of metal oxide nanomaterials can be elucidated.

## Mechanistic insight into the assembly of POVs

Recent efforts in materials science have focused on reducing the size of bulk materials to their nanoscopic congeners. Synthetic control of the formation of these nanocrystals (NCs) is of importance due to the implications of size, phase, and morphology on the physicochemical properties of these materials.^54–57^ Among various synthetic routes to form metal oxide NCs, liquid phase synthesis is a widely used method, as it employs reactive homogeneous precursors to template the formation of the assembly. This method cuts off nucleation from the growth stage of NC formation, which helps to control the particle size and reproducibility.^58,59^

In these self-assembly synthetic methodologies, several factors such as reaction time, temperature, pH of the solution, concentration of capping ligands, proper choice of the solvent, use of supercritical fluids,^60–62^*etc.* influence the shape, crystallinity, monodispersity, size, and quality of the NCs. Furthermore, solvothermal synthesis in organic solvent allows for the exploitation of the properties of added ligands to control the reactivity of the precursor, directly impacting nucleation and growth of the NCs. Despite this seemingly enhanced control of NC properties *via* solvothermal synthesis, the ‘black box’ nature of these reactions often acts as a barrier for understanding the atomistic mechanism of the self-assembly of molecular precursors. This translates to challenges in the scale up of solution-state syntheses of NCs. Thus, mechanistic insight into the reaction is of critical importance for achieving particular phases and morphologies of nanoscopic materials.^63^

As described above, POMs have been touted as structural models for bulk metal oxides. Both the elemental composition and physicochemical properties of POMs have been cited as broadly analogous to their nanocrystalline counterparts. Like the solvothermal and self-assembly mechanisms invoked for NC formation, the careful manipulation of reaction conditions allows for control over the resultant structure of a POM. As POM chemistry has developed, a variety of nanometer-scale, molecular structures have been discovered. While the stability of these metal–oxo architectures has been well established, significantly less is known about the mechanisms by which these complexes form.^23,64^ Insights into the self-assembly pathways of POMs are extremely limited in the literature. Only recently the mechanism of formation of the molybdenum oxide cluster (Mo_132_: Mo^VI^_72_Mo^V^_60_O_372_(CH_3_COO)_30_(H_2_O)_72_]^42−^)^65,66^ has been reported. Further, Bo and co-workers have developed a computational method that can predict the mechanism of metal oxide cluster (octamolybdate) formation based on the pH, temperature, ionic force, and its speciation model, which defines notable steps in the nucleation process.^67^ The methodology helped them to solve non-linear equations involving multiple equilibria to point out which of these matches well with the experimental data to predict the formation mechanism automatically.^67^ Collectively, these reports demonstrate the importance of understanding the step-by-step formation of inorganic clusters, as this work will ultimately enable the generation of structurally diverse POMs with targeted properties.

Researchers interested in the formation of POVs have used a variety of techniques to generate these clusters. Through the modulation of the multidimensional concentration/pH diagram of vanadium–oxo species and reaction scales, it has been possible to control the distribution of the resultant POV clusters in solution.^68^ The formation of POVs is generally thought to proceed through the self-assembly of monometallic vanadium oxide molecular precursors. Specific vanadium precursors have been used to generate Keggin, Lindqvist, or spherical POV structures with mixed-valent (V^IV^/V^V^) metal centres.^51,69^ Despite a large number of reported POV assemblies, only a handful of publications have tackled the challenge of elucidating the mechanism of formation of these clusters.^28,36–40,47,70–73^

### Mechanistic insight into the aqueous formation of POVs

**Fig. 2:**
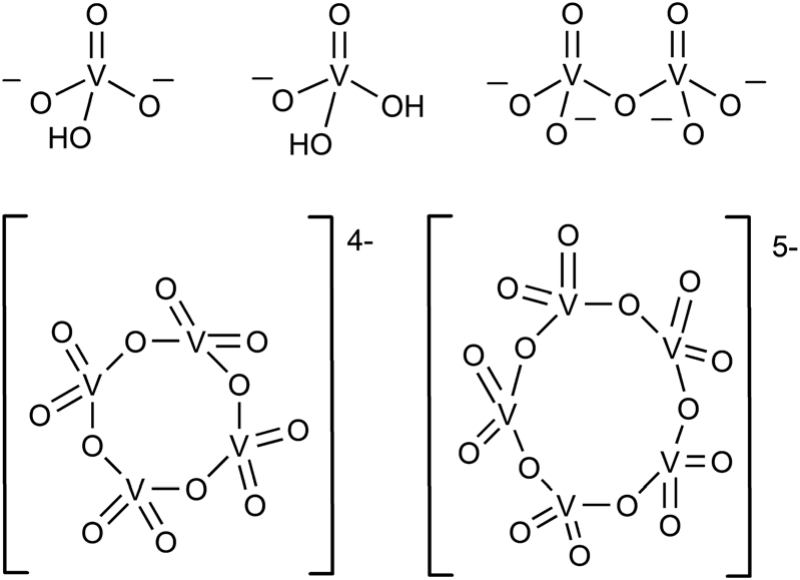
The representative structures of [HVO_4_]^2−^, [H_2_VO_4_]^1−^, dimeric [V_2_O_7_]^4−^, tetrameric [V_4_O_12_]^4−^, and pentameric [V_5_O_15_]^5−^.

In the aqueous synthesis of POVs, pH plays a vital role in dictating self-assembly pathways. The formation of polynuclear POV assemblies in water was investigated by Westra *et. al.* using ^51^V NMR and electronic absorption spectroscopy.^74^ In this work, the authors interrogated the chemistry of the orthovanadate molecular building unit [VO_4_]^3−^ (Fig. 2), which is stable in alkaline conditions (pH = 13–14). Decreasing the pH of an aqueous solution of [VO_4_]^3−^ results in the formation of a series of proposed intermediates: [HVO_4_]^2−^, [H_2_VO_4_]^1−^, H_3_VO_4_, as well as [VO_2_(H_2_O)_4_]^1+^.^74^ The formation of terminal “V–OH” and “V–OH_2_” moieties upon acidification of the vanadium oxide subunit triggers a series of condensation reactions that result in the generation of bridging oxido ligands, and ultimately, a variety of polynuclear architectures. For example, at low concentration and neutral pH, the monomeric [H_2_VO_4_]^1−^ and [HVO_4_]^2−^ species were found to react with one another to form [V_2_O_7_]^4−^, which subsequently is converted to the tetrameric ([V_4_O_12_]^4−^) and pentameric ([V_5_O_15_]^5−^) assemblies.^75,76^

Westra and co-workers subsequently reported calorimetric studies on this system, which shed light on the thermodynamics of oligomerization of vanadium oxide moieties (pH ≥ 8, 1 M NaClO_4_).^77^ The authors reported the enthalpically favoured dimerization of [HVO_4_]^2−^ species to [V_2_O_7_]^4−^ (Δ*G*^0^ = −5 kJ mol^−1^), accomplished *via* condensation of two discreet vanadate units. The dimerization triggered restriction in the freedom of species owing to negative entropy contribution. Further proton assisted addition of subsequent HVO_4_^2−^ to [V_2_O_7_]^4−^ to form [V_3_O_10_]^5−^ (Δ*G*^0^ ([V_3_O_10_]^5−^) = −70 kJ mol^−1^) and [V_4_O_13_]^6−^ (Δ*G*^0^ ([V_4_O_13_]^6−^) = 135 kJ mol^−1^) stabilized the growth of higher nuclear structures, suggesting favourable energetic contributions for condensation reactions following the addition of protons to the V–O bond. Moreover, the preferred formation of the stable cyclic structure [V_4_O_12_]^4−^ (Δ*G*^0^ ([V_4_O_12_]^4−^) = −239 kJ mol^−1^) over the linear tetramer [V_4_O_13_]^6−^ in acidic environments offers addition support for proton-dependent speciation of clusters in aqueous solution.

Additional insight into the effects of acid (*i.e.* sulfuric acid) on the stability of the local hydration structures of vanadyl ions (*e.g.* VO^2+^, and VO_2_^+^) was garnered through *ab initio* studies.^78^ Interestingly, the protons were not found to react with the VO^2+^ cation. This result contrasted with that of the reactivity of VO_2_^+^ with sulfuric acid, in which case protonation of an oxo moiety was observed. It is important to note that selective protonation of high-valent vanadyl moieties promotes requisite condensation steps for the formation of bridging oxo ligands (V–O–V) through the elimination of water. This provides theoretical support for the formation of “V–OH” and “V–OH_2_” moieties upon acidification, which supports stitching of smaller “V–O” fragments to form larger architectures.

Exposing similar trends in the stability of POV clusters, Rompel and co-workers have reported on the stability of plenary POV motifs.^79^ This work has bolstered support for the observation that pH has a strong effect on cluster stability, and dictates the size and shape of POV assemblies. At pH > 7, [V_10_O_28_]^6−^ is converted to lower-order assemblies; first, conversion to the tetrameric vanadium oxide cluster is observed [V_4_O_13_]^6−^ (pH ∼ 8–10), which progresses to the dimeric [V_2_O_7_]^4−^ (pH ∼ 10.5–12.5) and monomeric [VO_4_]^3−^ (pH ∼ 13–14) species. It is worth noting that the conversion to the decavanadate ion to lower-order POVs is dependent also on vanadium concentration, temperature and ionic strength.^80^

Throughout multiple reports, the pH dependant stability of POVs and the mechanistic insights into how individual vanadate moieties link together through oxide bridges to generate [V_4_O_12_]^4−^ and [V_5_O_15_]^5−^ have been established.^81,82^ However, it is interesting to note that there are no reports of isolated POVs from aqueous reaction medium containing six to nine vanadium centres. This has been justified by the tendency of the vanadium–oxo clusters to form closed shell spherical structures in water, composed of several fused polyhedra or by linking common vertexes. The decavanadate ion [V_10_O_28_]^6−^, and its various protonated forms, are the major species found in the pH range 2 to 6. Notably, this cluster possesses vanadium centres with unusual octahedral coordination geometries. For more information on the stability of POVs across various oxidation states in water or organic solvent, the readers are encouraged to read articles by Westra and Hayashi.^77,83^

### Mechanistic insight into the nonaqueous formation of POVs

The self-assembly of POVs in organic solvent presents a new set of challenges in considering the mechanism of vanadium aggregation due to the absence of hydrolytic pathways which trigger condensation reactions to form POVs in water. Thus, in organic solvent, the majority of studies have focused on the use of polynuclear vanadium oxide clusters as ‘seeds’ to promote POV growth. By comparison, little is known about the formation of precursors from a single vanadium centre. The relevance of non-aqueous solvent in POV formation is twofold, (i) it provides access to new POVs which were not observed in water,^39^ (ii) it prevents the rapid polymerization of smaller building blocks due to the absence of proton triggered condensation pathways.

**Fig. 3:**
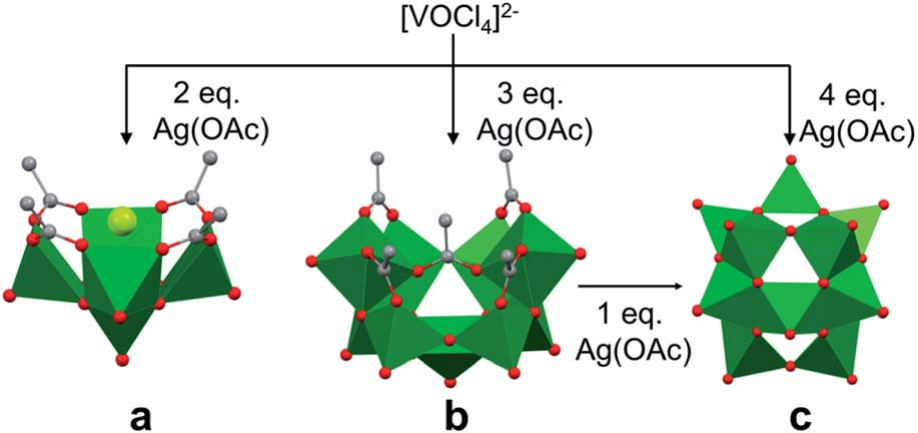
Crystal structures of (a) [V_5_O_9_Cl(OAc)_4_]^2−^, (b) [V_9_O_19_(OAc)_5_]^3−^ and (c) [V_15_O_36_]^5−^. Colour code: V: green polyhedra, O: red sphere, Cl: light green sphere, C: grey sphere. Hydrogen atoms, solvent molecules, and counter cations are omitted for clarity.

Christou and co-workers reported the mechanism of self-assembly of vanadium oxide clusters in organic solvent, highlighting the authors' ability to synthesize and characterize individual [V_5_O_9_Cl(OOCCH_3_)_4_]^2−^, [V_9_O_19_(OOCCH_3_)_5_]^3−^ and [V_15_O_36_]^5−^ clusters by varying the reactant ratios (*e.g.* [VOCl_4_]^2−^: AgOOCCH_3_ (AgOAc); 1 : 2, 1 : 3 and 1 : 4) in acetonitrile (Fig. 3).^84^ Here, the acetate ligands function as capping agents, while the chloride ion serves as a template for cluster assembly. The choice of silver acetate as an oxidant was crucial, as it served as both the acetate source and a reagent poised for chloride abstraction (*e.g.* precipitation of AgCl from acetonitrile). The acetate capping ligand in [V_5_O_9_Cl(OAc)_4_]^2−^ dissociates under the reaction conditions, providing unsaturated vanadium coordination spheres which are subsequently occupied by smaller ‘V–O’ fragments, resulting in the formation of clusters of higher nuclearity, *i.e.* [V_9_O_19_(OAc)_5_]^3−^ and [V_15_O_36_]^5−^.

Due to structural similarities, [V_9_O_19_(OAc)_5_]^3−^ was considered to be an extension of [V_5_O_9_Cl(OAc)_4_]^2−^. The authors credit the carboxylate ligands for playing an important role in preventing further aggregation (Fig. 3). The presence of a common {V_7_O_17_} unit in both the [V_9_O_19_(OAc)_5_]^3−^ and [V_15_O_36_]^5−^ structures, and the formation of the carboxylate-free [V_15_O_36_]^5−^ core from the re-dissolved crystals of [V_9_O_19_(OAc)_5_]^3−^ in acetonitrile with the addition of an additional equivalent of silver acetate, further supports the hypothesis of seeded growth.^84^ It is important to note that the authors were unable to determine the mechanism by which vanadium centres aggregate to form the smallest POV assembly (*e.g.* [V_5_O_9_Cl(OAc)_4_]^2−^).

A large number of reported POV architectures have been accessed using the [V_10_O_28_]^6−^ cluster as a ‘seed’ under various reaction conditions (*e.g.* [V_12_O_32_]^4−^,^7^ [V_13_O_34_]^6−^,^8^ [V_17_O_42_]^4−^ and [V_6_O_13_{(OCH_2_)_3_CR}_2_]^2−^ [R = NO_2_, CH_2_OH, CH_3_]).^85–87^ While this route had proved to be an effective strategy for accessing a diverse library of POV clusters, mechanistic insight into the formation of these assemblies from [V_10_O_28_]^6−^ has not been investigated. It was hypothesized that refluxing a solution of [V_10_O_28_]^6−^ results in transient access to dynamic substructures of the parent ion through the cleavage and regeneration of V–O bonds.

**Fig. 4:**
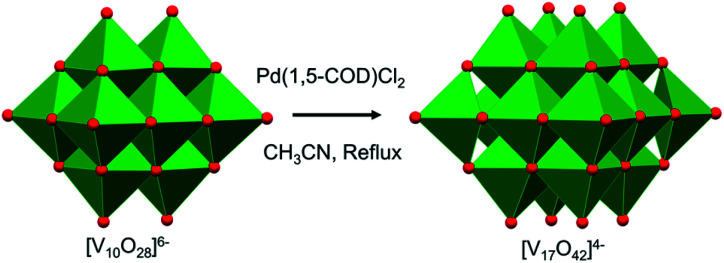
Reductive coupling of [V_10_O_28_]^6−^ with Pd(1,5-COD)Cl_2_ to form [V_17_O_42_]^4−^. Colour code: V: green polyhedra, O: red.

More recently, mechanistic work invoking the use of the decavanadate ion as a ‘seed’ for the formation of POV clusters has been reported by Hayashi and co-workers. Termed as the “reductive coupling method”, the authors note that refluxing (^*n*^Bu_4_N)_3_[H_3_V_10_O_28_] in an organic solvent in the presence of a coupling agent, [Pd(1,5-COD)Cl_2_], results in the formation of (^*n*^Bu_4_N)_4_[V_17_O_42_] (Fig. 4).^88,89^ They proposed the following three steps as the mechanism of formation of the large POVs: (1) the reaction of [Pd(1,5-COD)Cl_2_] (COD: cyclo-octadiene) with (*n*-Bu_4_N)_3_[H_3_V_10_O_28_] produces a Pd–POV supported complex;^90^ (2) at high temperature, acetonitrile facilitates oxidation of the organometallic species by the POV, resulting in the formation of a reduced vanadium oxide cluster, and finally (3) the spontaneous coupling between the reduced POV and the initial cluster, [V_10_O_28_]^6−^, gives rise to the formation of larger vanadium oxide architectures. These examples provide an additional pathway by which POV architectures can be manipulated to break apart and reform into larger, more complex systems, the control of which can enable researchers to make new molecular systems with desired properties.

**Fig. 5:**
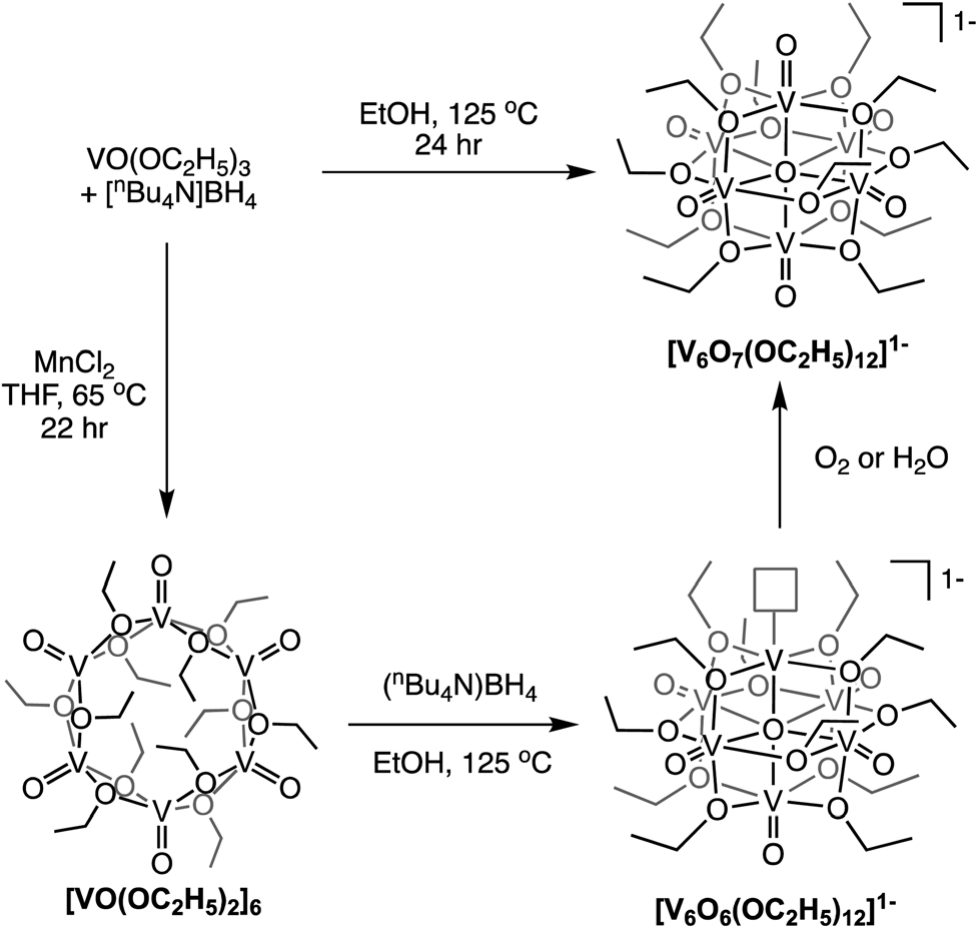
Conversion of a cyclic [VO(OC_2_H_5_)_2_]_6_ cluster to the Lindqvist structure under solvothermal conditions.

Finally, Matson and co-workers reported the synthesis of a cyclic, low-valent POV–alkoxide cluster, [VO(OC_2_H_5_)_2_]_6_, and its subsequent conversion to an oxygen-deficient, Lindqvist assembly [V_6_O_6_(OC_2_H_5_)_12_]^*n*^ (*n* = 1–, 0).^91^ At low temperature, formation of the cyclic {V_6_} architecture, consisting of six edge-sharing {VO_5_} fragments, was observed. In this structure, ethoxide ligands occupy all bridging positions (Fig. 5). It was proposed that the formation of the cyclic [VO(OC_2_H_5_)_2_]_6_ cluster by stitching together reduced “VO(OC_2_H_5_)_2_” fragments was templated by chloride anions present in the solution.^92^ Notably, the authors demonstrated that under solvothermal conditions, the cyclic cluster, [VO(OC_2_H_5_)_2_]_6_, can be converted to the thermodynamically stable Lindqvist assembly [V^III^V^IV^_4_V^V^O_6_(OC_2_H_5_)_12_]. These results suggest that the reduction-triggered polymerization of vanadyl alkoxide precursors serves as a key step in the formation of the Lindqvist clusters.

Collectively, the sample of reports summarized here offer insight into the use of molecular precursors for the formation of discrete cluster architectures. However, there remains a great deal of work to be done to understand explicitly how individual vanadium atoms interact in solution to manifest in the formation of nanoscale POVs. In aqueous solution, formation of different POV architectures triggered by the pH of the solution and condensation reactions between smaller vanadium oxide units has been explored, but targeted formation of POVs with specific nuclearity remains unrealized. In organic solvent, templated synthesis of a POV structure provides mechanistic insight for the formation of POVs of particular nuclearity and shape. Still, the role of adventitious water in the formation of these clusters in organic medium remains unclear. It is clear that additional studies are necessary to further develop a proper mechanistic understanding of vanadium oxide cluster formation.

## Organic functionalization of polyoxovanadates: modelling ligand/core interactions in nanoscopic assemblies

The influence of organic surface ligands on the structure, properties, and reactivity of metal oxide NCs has been widely explored in the field of materials science. These organic moieties serve to tune particle size, structure, monodispersity, and stability, as well as the solubility, reactivity (*i.e.* surface exposure), and the electronic properties of the metal oxide. Collectively, experimental observations demonstrate a clear correlation between ligand composition and the performance of a nanocrystalline material in a variety of applications (*e.g.* biomaterials, photoactive materials, and electronic devices).^93–96^ Where an extensive library of nanocomposites has been studied and their properties experimentally defined, less is known about the atomistic ligand–particle interactions which produce the observed properties. To better understand these important characteristics of nano assemblies, researchers have turned to the study of molecular cluster complexes as model systems.

Functionalization of polyoxometalates with organic ligands has resulted in the generation of a variety of hybrid compounds. Insertion of organic ligands to both bridging and terminal sites of the metal oxide core has enabled researchers to add siloxide, phosphonate, carboxylate, and alkoxide moieties to the cluster surface, imparting new physicochemical properties to the metal oxide core. For an expansive view of the development and applications of POM–organic hybrids, the readers are referred to the following relevant review articles.^97–102^

**Fig. 6:**
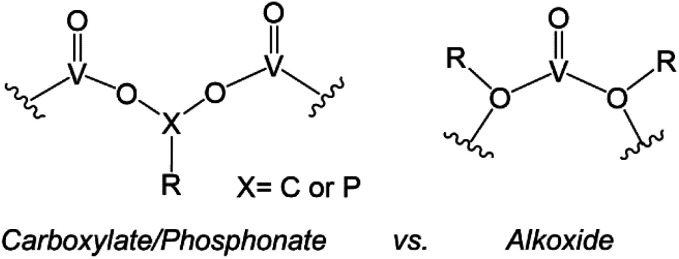
Binding motifs of carboxylates and phosphonates (left), and alkoxides (right) in POV cluster frameworks.

Similarly, the organofunctionalization of POVs has been reported with carboxylate^103–107^ and phosphonate^108,109^ groups (Fig. 6). Carboxylate-functionalized POVs were first reported in 1976 by Mattes and co-workers; these vanadium oxide assemblies feature bidentate carboxylate moieties that decorate the periphery of the cluster core, yielding open, basket-shaped structures.^110^ More recently, surface functionalization of POV clusters has been extended to phosphonate derivatives, taking advantage of a similar binding motif to lower-nuclearity POV–carboxylates. In both cases, the cluster framework is formed from mononuclear precursors in the presence of the organic ligand. Due to the high diversity of carboxylate- and phosphonate-containing organic molecules available, supramolecular structures based on POV building blocks, such as cages,^105–107^ rings,^104^ and capsules,^108,109^ have all been reported. The size and shape of these structures can be tuned with a high degree of precision, allowing researchers to envision applications of these materials in chemical separation through host–guest interactions.

The aforementioned examples of organofunctionalized POVs represent intriguing examples of these hybrid nanoassemblies; however, these systems are significantly less studied in comparison to alkoxide-functionalized vanadium oxide clusters. The generation of polyoxovanadate–alkoxide (POV–alkoxide) clusters can be accomplished by either self-assembly of mononuclear precursors or pre-synthesized cluster intermediates (*e.g.* [V_10_O_28_]^6−^), or alternatively through post-synthetic incorporation of organic groups *via* condensation. The former synthetic pathway accounts for the most reports detailing POV–alkoxide assembly (*vide infra*). The binding motif of alkoxide groups varies quite distinctly from that of carboxylates and phosphonates, resulting in bridging alkoxide links (V–O–V) within the core, as opposed to V–O–X–O–V (X = C, P) linkages on the periphery (Fig. 6). This in turn yields compounds which do not typically adopt open frameworks, translating to a reduced diversity in cluster core structures. Given that these complexes have been the subject of thorough investigations regarding both their structural and electronic properties, we have elected to focus this overview on POV–alkoxide clusters, elaborating on their ability to serve as atomically precise models for rationalizing the influence of organic functional groups on the physicochemical properties of nanocrystalline metal oxide materials.

### Distortion of the POV core *via* surface decoration with rigid, multidentate ligands

**Fig. 7:**
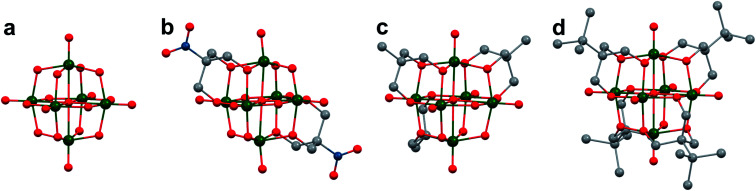
Molecular structures of Lindqvist-type POV: (a) [V_6_O_19_]^8−^; (b) [V_6_O_13_(TRIS^CH_3_^)_2_]^2−^; (c) [V_6_O_10_(TRIS^CH_3_^)_3_]^1−^; and (d) [V_6_O_7_(TRIS^*t*Bu^)_2_]^2−^. Colour code: V: green, O: red, C: grey, N: blue. Hydrogen atoms, solvent molecules, and counter cations have been omitted for clarity.

Whereas Lindqvist-type polyoxotungstates and polyoxomolybdates are stable as fully-inorganic clusters (*i.e.* composed solely of transition metals and oxide ligands), researchers have found that the isolation of the vanadium-based congener requires incorporation of alkoxide ligands at the periphery of the assembly (Fig. 7). The presence of these functional groups reduces the overall charge of the hexavanadate assembly, thus stabilizing the small ionic radius of the vanadyl ions. An early example of alkoxide-bridged, Lindqvist-type POV was reported by Zubieta and co-workers in 1992.^86^ This complex, which resulted from the reaction of a decavanadate cluster [H_3_V_10_O_28_]^3−^ and an excess of a tris(hydroxymethyl)methane (TRIS) capping ligands, features a Lindqvist V^V^ oxide core with two capping groups located on opposing ends of the cluster^86^ (*i.e.* [V_6_O_13_((OCH_2_)_3_CR)_2_]^2−^; R = NO_2_, CH_2_OH, NMe_2_, CH_3,_ C_2_H_5_).^86^ This cluster framework has since been the focus of significant synthetic modification which makes it particularly intriguing for studying the influence that capping groups have on nanocrystalline materials. For example, Zubieta and co-workers were able to access Lindqvist POV–alkoxides with three ([V_6_O_7_(OH)_3_((OCH_2_)_3_CCH_3_)_3_]^1−^) and four ([V_6_O_7_((OCH_2_)_3_CCH_3_)_4_]^2−^) tridentate ligands.^111–114^

While typical Lindqvist POMs feature highly symmetrical metal–oxo units, the introduction of capping tripodal ligands significantly reduces the molecular symmetry of the Lindqvist core. The six bridging oxo groups and six bridging alkoxo groups in [V_6_O_13_((OCH_2_)_3_CR)_2_]^2−^ have average V–O_b_ bond lengths of 1.823 Å and 2.022 Å, respectively (Fig. 7b, Table 1). In comparison, the rhodium-capped cluster, [(Cp*Rh)_4_V_6_O_19_], a rare example of an assembly with a ‘[V_6_O_19_]^8−^’ core, all bridging positions are occupied by μ^2^–oxo moieties, resulting in equivalent V–O bond lengths of 1.914 Å (Fig. 7a, Table 1).^115–117^ The bond perturbations in the POV–alkoxide showcase that functionalization of the bridging oxos results in lattice distortions, with a ∼0.1 bond truncation of the V–O–V moieties as a consequence of the rigidity of the organic ligands. Changes to the core structure of the Lindqvist assembly are most pronounced in analysis of the Ot–V–O_c_ bond angle (O_t_ = terminal oxido ligand; O_c_ = m_6_-oxido moiety positioned at the centre of the cluster). In a purely symmetrical structure, such as [(Cp*Rh)_4_V_6_O_19_], this angle is a perfectly linear 180°, as all the VO_6_ units are equivalent.^115,116^ Installation of two TRIS capping groups significantly distorts the core, contracting this angle by an average of 8.36°, observed as a “zig-zag” across the centre of the cluster.^86^

**Table 1:** Solid state structural parameters of Lindqvist POVs with varying degrees of organofunctionalization

Complex	V–O_b_ (Å)	V–O(R) (Å)	V–O_c_ (Å)	O_t_–V–O_c_
[V_6_O_19_]^8−^	1.914	—	2.246	180°
[V_6_O_13_((OCH_2_)_3_CR)_2_]^2−^	1.823	2.022	2.244	171.64°
[V_6_O_10_((OCH_2_)_3_CR)_3_]^1−^	1.999	1.973	2.328, 2.272	179.29°
[V_6_O_7_((OCH_2_)_3_CR)_4_]^2−^	—	2.07	2.315	180°
[V_6_O_13_(OCH_3_)_6_]^2−^	1.834	1.989	2.236	172.92°
[V_6_O_12_(OCH_3_)_7_]^1−^	1.868	1.974	2.243	173.58°
[V_6_O_11_(OCH_3_)_8_]^2−^	1.818	1.952	2.233	174.20°
[V_6_O_8_(OCH_3_)_11_]^+^	1.821	1.962	2.274	177.60°
[V_6_O_7_(OCH_3_)_12_]^1−^	—	1.990	2.305	177.96°

Increasing the number of TRIS ligands occupying surface sites of the vanadium oxide cluster results in expansion of the metal–oxide core; incorporation of three TRIS functionalities results in an increase in V–O bond lengths of 0.085 and 0.059 Å for bridging oxo and alkoxo linkages, respectively (Fig. 7c; Table 1).^114^ In addition, the distance of the vanadium ions to the central oxygen atom (O_c_), was observed to increase non-uniformly by 0.082 and 0.26 Å for vanadium centres with both bridging oxos and alkoxides, and ions containing exclusively alkoxide ligands, respectively. The sites containing unconstrained bridging groups were forced outward to relieve the internal pressure on the core, leading to longer V–O_c_ bonds. Increased incorporation of rigid groups holds the core in a more symmetrical configuration, as evidenced by the transition of the O_t_–V–O_c_ angle toward linearity (179.29°) (Table 1).^114^ The fully-functionalized [V_6_O_7_((OCH_2_)_3_CCH_3_)_4_]^2−^ features further inflammation of the Lindqvist core, with both the V–O_b_ and V–O_c_ distances being elongated by 0.156 and 0.069 Å, respectively (Fig. 7d, Table 1).^114^

Single crystal X-ray diffraction of these atomically precise, alkoxide-functionalized vanadium oxide assemblies reveals structural consequences of surface functionalization with multidentate alkoxide ligands on extended metal oxide framework. Rigidity of the organic ligands manipulates the local geometry of the assembly, pushing, pulling, and expanding the framework to accommodate the capping group.

**Fig. 8:**
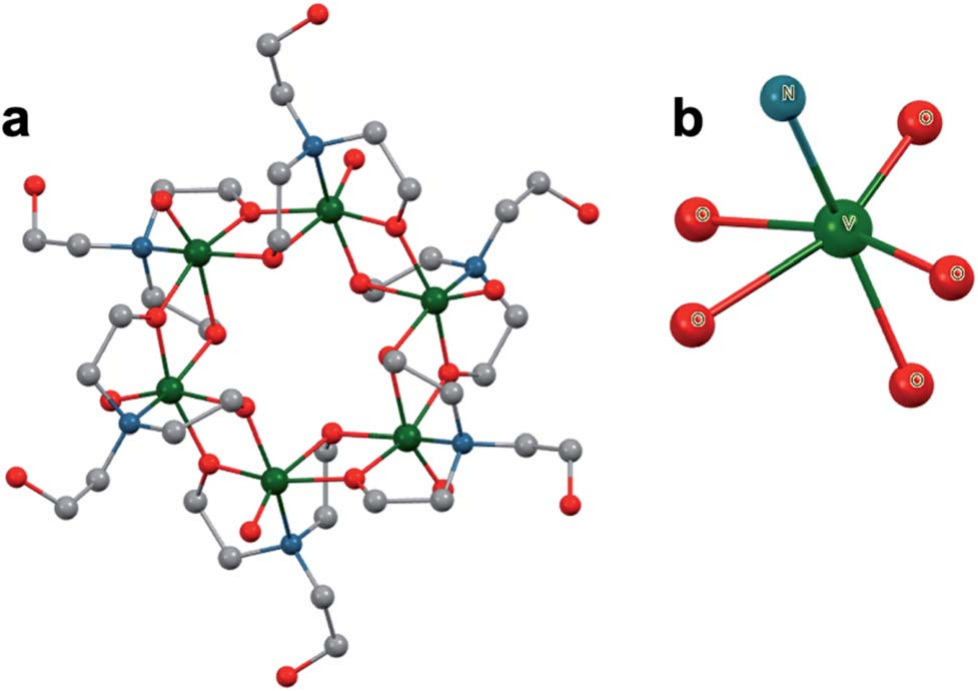
(a) Molecular structure of an Anderson-type POV–alkoxide cluster, [V_6_O_6_{(OCH_2_CH_2_)_2_N(CH_2_CH_2_OH)}_6_], featuring triethanolamine bridging ligands; and (b) the ligand environment about each V^IV^ ion in the molecule. Colour code: V: green, O: red, C: grey, N: blue. Hydrogen atoms, solvent molecules, and counterions omitted for clarity.

Another example featuring the functionalization of the decavanadate POV cluster with multidentate alkoxide ligands was reported by Khan and co-workers in 2006, where exposure of [H_3_V_10_O_28_]^3−^ to di- and triethanolamine under solvothermal conditions produced the Anderson-type POV: [MV^IV^_6_O_6_{(OCH_2_CH_2_)_2_N(CH_2_CH_2_OH)}_6_]^*n*^ (*n* = +1; M = Li, Na & *n* = +2; M = Mg, Mn, Fe, Co, Ni), with all six vanadium centres in the V^IV^ oxidation state (Fig. 8a).^118–120^ The disparity in cluster structure between Zubieta and Khan's complexes is likely due to ligand rigidity differences, with the rigid TRIS ligands unable to accommodate the ring structure, and the ethanolamine ligands' increased flexibility favouring the less sterically confined Anderson framework. The observed tridentate binding motif arises from a VO_5_N ligand sphere, with a single terminal oxo, four bridging alkoxo, and a capping N group (Fig. 8b).^120^ Two of the alkoxo groups are from the same ligand as the bound N; however, the other two are from the ligands of the neighbouring V ion, which gives rise to the cyclic framework. This distorted octahedral ligand environment, which is a consequence of the nature of these conformationally flexible organic moieties, disallows the formation of the Lindqvist core. These results are notable given the correlations between POM properties and the type of metal oxide structure; the geometry, M–O bond lengths, magnetic moments, vibrational modes (IR, Raman), and charge transfer characteristics are all influenced by the shape of the assembly. The results from this work illustrate how the chemical makeup of a capping ligand can dictate the core electronic properties of the resultant molecular assembly.

Overall, these investigations highlight the ability of POV–alkoxides to tolerate changes to the surface ligand environment. Ligand rigidity and quantity play a role in modulating the core structure and symmetry of these clusters, even resulting in the formation of unexpected framework geometries. This can be directly monitored *via* analysis of crystal structure parameters. By selecting a specific capping ligand, particular core structures may be selectively accessed, precisely generating complexes with desired core properties.

### Synthetic methods for the generation of POV–alkoxides with monodentate surface ligands

**Fig. 9:**
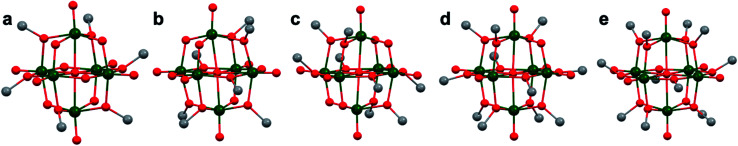
Molecular structures of Lindqvist-type POV: (a) [V_6_O_13_(OCH_3_)_6_]^2−^; (b) [V_6_O_12_(OCH_3_)_7_]^1−^; (c) [V_6_O_10_(OCH_3_)_8_]^2−^; (d) [V_6_O_8_(OCH_3_)_11_]^1+^ and (e) [V_6_O_7_(OCH_3_)_12_]^1−^. Colour code: V: green, O: red, C: grey, N: blue. Hydrogen atoms, solvent molecules, and counter cations are omitted for clarity.

An addition class of organofunctionalized polyoxovanadates includes those that are bridged by monodentate alkoxide ligands of the general form, [V_6_O_19−*x*_(OR)*_x_*] (*x* = 6, 7, 8, 11, 12; Fig. 9).^46,121–123^ These POV–alkoxides have been isolated by various synthetic approaches. The first isolated complex in this series was the heptamethoxy-substituted [V_6_O_12_(OR)_7_]^1−^ reported by Hill and co-workers (Fig. 9b).^122^ This complex is synthesized by similar conditions to [V_6_O_13_(TRIS)_2_]^2−^; refluxing the decavanadate precursor in methanol for 48 hours. Interestingly, where one would expect only six methoxide moieties would be present on the core, adopting a molecular structure analogous Zubieta's TRIS-functionalized complexes, [V_6_O_13_(TRIS^R^)_2_]^2−^, seven bridging moieties are alkoxylated, decreasing the overall charge of the cluster to a monoanion. This also results in significant reduction in cluster symmetry and therefore structural distortions as a consequence of the odd number of alkoxide surface ligands (Table 1).

The hexamethoxo analogue, [V_6_O_13_(OCH_3_)_6_]^2−^, was subsequently reported by Krebs and co-workers *via* self-assembly of VO(acac)_2_ (acac = acetylacetonate) in methanol in the presence of bis(1-methylimidazol-2-yl)-4-methoxyphen-1-ylmethanol (bmimpm) and triethylamine (Fig. 9a).^46,122^ In contrast to the other systems reported thus far, the resultant complex also features coordination of two VO(bmimpm)(acac) moieties bound to the cluster at terminal oxo positions on opposing termini of the framework. The vanadium ions comprising the cluster are in the 5+ oxidation state, confirmed by bond valence sum calculations and electrochemical measurements. This means that the V^IV^ ions of the VO(acac)_2_ precursor are oxidized during synthesis. The methoxide ligands are located at more symmetrical positions, where two bridging sites of all the core vanadate units are alkoxylated, yielding uniform structural perturbations in the ligand field of all six vanadium centres (Table 1).

The next monodentate-alkoxide-functionalized POV cluster reported was a fully-alkoxylated Lindqvist cluster: [V_6_O_7_(OCH_3_)_12_]^1−^ (Fig. 9e).^124^ Hartl and co-workers generated this complex *via* a solvothermal reaction of VO(O^*t*^Bu)_3_ and [^*n*^Bu_4_N]BH_4_ in methanol.^124^ A similar complex, which features bridging ethoxide units, was later synthesized by an analogous procedure, substituting ethanol as the solvothermal reaction medium.^45^ Being that all twelve bridging positions are decorated with alkoxide ligands, a symmetrical Lindqvist core was produced (Table 1). This complex features a mixed-valent core, with an oxidation state distribution of [V^IV^_5_V^V^].

In 2009, Hartl and co-workers reported the molecular structure of [V_6_O_8_(OCH_3_)_11_]^1+^, isolated as a byproduct in the original synthetic procedures for formation of [V_6_O_7_(OCH_3_)_12_]^1−^ (Fig. 9d).^46^ Being that the complex is nearly entirely alkoxylated, the observed structural perturbations deviate only slightly from its fully-alkoxylated congener (Table 1).

Most recently, the serendipitous discovery of the octomethoxo-functionalized cluster, [V_6_O_11_(OCH_3_)_8_]^2−^, was accomplished as a counter ion to a Co-scorpionate species.^123^ This is accomplished by heating a mixture of Co powder, VOSO_4_, NH_4_SCN, and 1-hydroxymethyl-3,5-dimethylpyrazole (L) in methanol for 5 hours. The first crop of crystals contains [Co(L)SCN]_2_[V_6_O_11_(OCH_3_)_8_] (Fig. 9c). With the four unfunctionalized bridging oxos located at sites which form a mirror plane through the central oxygen, the cluster features increased symmetry and only slight deviation from the ideal Lindqvist core (Table 1).

Investigations of the oxidation state distributions of the above hexanuclear cores reveals that, generally, increased alkoxide ligation results in the accumulation of reduced vanadium ions in these molecular assemblies. This is facilitated by the reduction in charge of bridging moieties from O^2−^ to O–R^1−^. Indeed, V^IV^ was not observed for either the hexa- or heptamothoxy functionalized POVs. The V^IV^ “threshold” for this series was found to be eight alkoxide moieties, which produce sufficient reduction in charge to stabilize mixed-valent cores. However, detailed electronic characterization of the hexa, hepta, and octomethoxo complexes are yet to be reported, so the ability of these complexes to stabilize further reduced states is unknown.

**Fig. 10:**
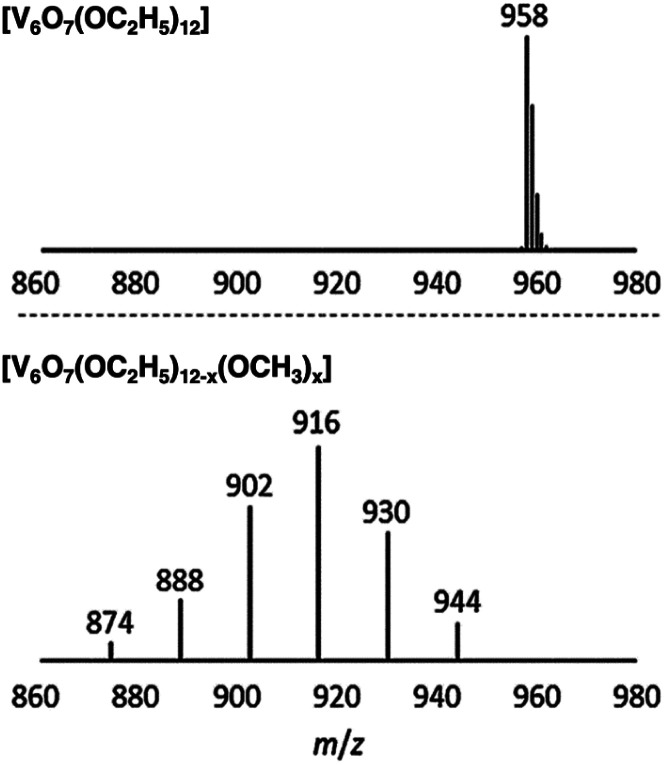
ESI-MS spectra of V_6_O_7_(OC_2_H_5_)_12_ (1-ethyl, top) and V_6_O_7_(OC_2_H_5_)_12−*x*_(OCH_3_)_*x*_ (*x* = 1–6) (2-ethyl, bottom). This figure has been adapted/reproduced from ref. 126 with permission from the Royal Society of Chemistry, copyright 2019.

Matson and co-workers were able to further expand the library of POV–alkoxide clusters through the use of propanol, butanol, pentanol, hexanol, 2-methoxyethanol (2-ME), and 2-ethoxyethanol (2-EE), as the solvent in solvothermal syntheses.^125–127^ It was observed that in using longer-chained alkoxide linkages, the phase of these complexes was changed (solid in the case of short alkoxide ligands, C1–C3, to an oil in the case of clusters with long, aliphatic alkoxide ligands, C5–C6).^125^ The generation of POV clusters that can be isolated as viscous oils is an interesting result, one which has an effect on the solubility of these complexes in organic solvents (*vide infra*). In subsequent reporting, the authors describe the synthesis of POV–alkoxide clusters with mixed alkoxide ligands, generating distributions of clusters with the general formula: V_6_O_7_(OR)_12−*x*_(OCH_3_)_*x*_ (R = C_2_H_5_ through C_6_H_13_, 2-ME, 2-EE).^126,127^ Notably, this synthetic approach does not generate a single mixed alkoxide compound, but a normal distribution of POV assemblies, as evidenced by electrospray ionization mass spectrometry (ESI-MS; Fig. 10). The degree of mixing is determined by assessing the visible masses to assign the generated combinations. Again, the material states were varied from solid (R = C_2_H_5_), to oils (R = C_4_H_9_ through C_6_H_13_, 2-ME, 2-EE). This range of POV–alkoxides reveal the robust stability of the Lindqvist core as well as provide a platform by which careful tuning of physical properties relevant for energy storage applications is accomplished. Analysis of the physical and electrochemical properties of this range of POV–alkoxides are detailed in subsequent sections (*vide infra*).

### POV–alkoxides with mixed-denticity pendant ligands as building blocks for POV-based materials, scaffolds for post-synthetic modification, and site-specific activity

**Fig. 11:**
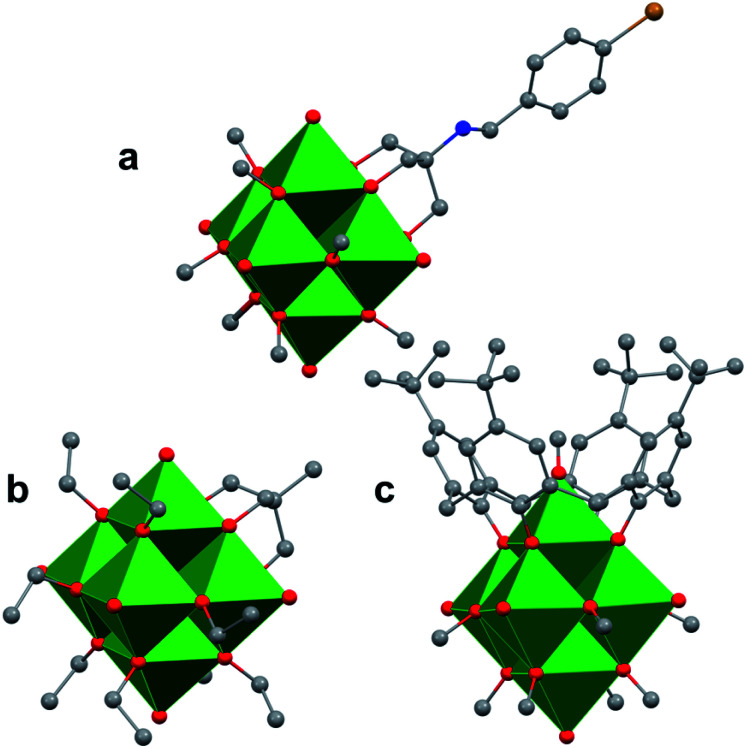
Molecular structures of mixed-dentate POV–alkoxide clusters: (a) [V_6_O_7_(OCH_3_)_9_TRIS^NC_7_H_6_Br^]^1−^; (b) [V_6_O_7_(OC_2_H_5_)_9_(TRIS^CH_3_^)]^1−^; and (c) [V_6_O_6_(OCH_3_)_8_(calix)]^1−^. Colour code: V: green polyhedra, O: red, C: grey, N: blue, Br: orange. Hydrogen atoms, solvent molecules, and counter cations are removed for clarity.

The study of POV–alkoxide clusters bearing bridging ligands of mixed denticity is a natural extension of the observation of the isolation of POV–alkoxide clusters with mono- and tri-dentate alkoxide ligands. Similar to that of the aforementioned tripod-ligated POVs first reported by Zubieta and co-workers, Spandl reported methods toward generating organically functionalized POVs featuring a single TRIS-derived functional group.^128^ Indeed, through addition of equimolar ratios of VO(O^*t*^Bu)_3_ and the TRIS^R^ ligands, the corresponding cluster, [V_6_O_7_(OCH_3_)_9_{(OCH_2_)_3_CR}] (R = CH_2_N_3_, NH_3_^+^), could be solvothermally synthesized. Following the generation of these organofunctionalized Lindqvist species, the authors demonstrated post synthetic modification of the functional group occupying the peripheral site of the TRIS^R^ ligand. In the case of the azide-terminated POV–alkoxide cluster, [V_6_O_7_(OCH_3_)_9_(TRIS^N_3_^)], functionalization of the cluster was accomplished *via* ‘Click chemistry’ in the presence of a variety of aromatic alkynes and Cu^I^(MeCN)_4_BF_4_. This resulted in the isolation of the triazole-functionalized POV–alkoxide clusters in good yield (Fig. 11a). The TRIS^NH^3+^^terminated POV–alkoxide cluster was condensed with a library of aryl aldehydes using B(OCH_2_CF_3_)_3_, producing an imine functionality on the pendant ligand. Similar types of post modifications have also been reported for nanoparticles, where introducing ligands with terminal carboxylic acid, amine, alcohol, alkene, alkyne, and azide groups provides reactive sites for additional functionalization.^129^

More recently, Matson and co-workers have extended this family of mixed-ligand complexes, reporting the synthesis of POV–alkoxide clusters featuring other TRIS-functionalized clusters. Tris(hydroxymethyl)ethane, as well as several other TRIS^R^ ligands (R = CH_2_OCH_3_, CH_2_OCH_2_OCH_3_, C_2_H_5_, NO_2_, N(CH_3_)_2_, and NC_5_H_4_) were employed in the synthesis of methoxide and ethoxide-bridged POV–alkoxides using adapted syntheses from their homoleptic congeners (Fig. 11b, R = CH_3_).^130,131^ Discussion of the effects of incorporation of tridentate ligands at the surface of POV–alkoxide clusters on cluster solubility and electronics is presented in a following section.

Comparison of Matson's V_6_O_7_(OC_2_H_5_)_9_(TRIS^R^) with Zubieta's TRIS-functionalized POV clusters reveals that the structural perturbations associated with the incorporation of two TRIS ligands are not observed in the mono-tripodal-functionalized cluster (Fig. 11b). This is due to the fact that, apart from the three bridging sites which are occupied by the TRIS ligands, the entire framework is bridged by ethoxide moieties. The presence of a single rigid tripod does not require asymmetric elongation of V–O_b_ bonds of the Lindqvist core to accommodate this group (*i.e.* the V–OR bond lengths and O_t_–V–O_c_ angles of the mono-TRIS complex are comparable to the homoleptic methoxide-bridged cluster, 1.993 Å *vs.* 1.990 Å and 177.43° *vs.* 177.96°, respectively).^86,124,130^

One final distinct entry to the family of mixed-ligand POV–alkoxide clusters was reported by Luneau and co-workers in 2008.^132^ Reaction of *p-tert*-butylcalix[4]arene (calix) with VOSO_4_ in the presence of a reductant in methanol yields a Lindqvist POV–alkoxide core, [V_6_O_6_(OCH_3_)_8_(calix)]^1−^ with one site-differentiated vanadate unit which is only bridged to neighbouring V ions through the calix ring (Fig. 11c). What's more, the site-differentiated V ion was found to be missing the expected terminal oxo ligand. Bond valence sum calculations revealed that this V ion is in the 3+ oxidation state. This molecule features highly intriguing magnetic properties; its magnetic moment is lower than one would expect for the case where all six V ions were independent, indicating exchange across the cluster core.

### Physical properties of POV–alkoxide clusters

Organic capping groups impart physical properties on nanocrystalline materials which are crucial for their use in their respective applications. Particle size, dispersity, and morphology have all been shown to be controlled by ligand identity.^93–95,133^ In addition, the chemical makeup of the capping ligands is known to influence particle dispersion in solution.^134,135^ This is critical to facilitate NC-substrate interactions, as mass transport limitations are minimized. Ligand density, or the number of capping groups per unit area on the NC surface, serves to both tune dispersion in a given solvent, as well as provide open sites for reactivity at metal centres. Alterations to ligand density provide a means for balancing solution dispersibility and material activity; precisely determining the limits of dispersion and ligand density are highly important for tuning the relevant physical properties of nanocrystalline systems to optimally perform in a desired application. A similar observation has been made with polyoxometalates, where long chain alkylammonium salts of clusters are necessary to dissolve these complexes in organic solvents such as acetonitrile (MeCN) or dichloromethane (DCM).

**Fig. 12:**
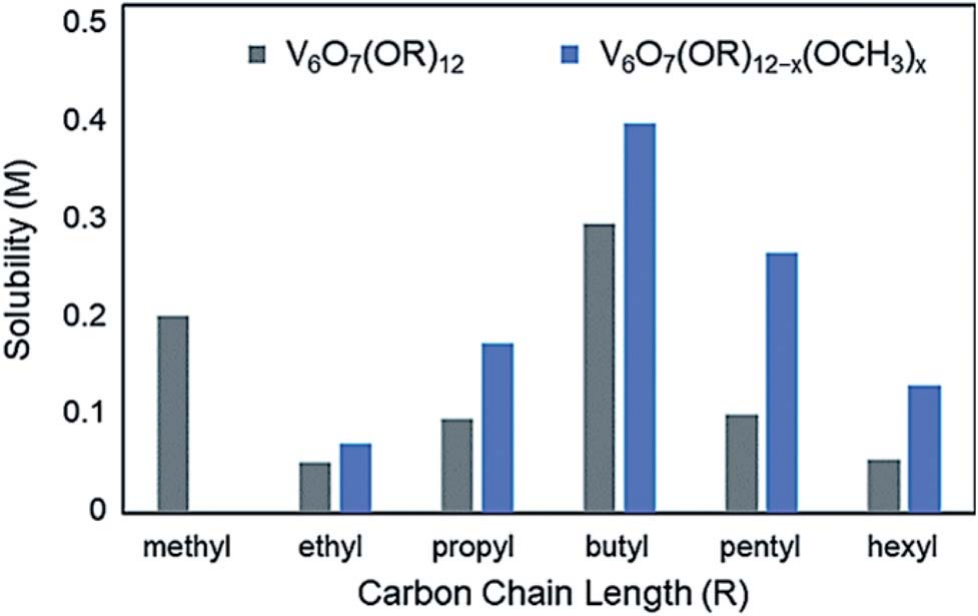
Solubility of homoleptic (grey) and heteroleptic (blue) aliphatic POV–alkoxide clusters in MeCN; this figure has been adapted/reproduced from ref. 126 with permission from the Royal Society of Chemistry, copyright 2019.

Similar results have been observed in the case of the aforementioned POV clusters functionalized with alkoxide ligands. Rigorous analysis of the change in solubility as a function of the identity of the bridging alkoxide ligand has been performed by Matson and co-workers in an effort to optimize this physical property of POV–alkoxide clusters for applications in flowable electrochemical energy storage.^136^ In MeCN, it was found that the butoxide-functionalized complex featured the highest solubility of the homoleptic, alkane-based alkoxide molecules (0.297 M),^125^ with the ethoxide-bridged complex being nearly insoluble in this solvent (0.052 M)^136^ (Fig. 12).

In a subsequent report, Matson and co-workers found that this cluster scaffold could tolerate mixed-alkoxide functionalization, generating distributions of clusters with the general formula: V_6_O_7_(OR)_12−*x*_(OCH_3_)_*x*_ (R = C_2_H_5_ through C_6_H_13_).^126^ For the full range of alcohols, solubilities of all generated mixtures were greater than their homoleptic congeners. This observation was attributed to a reduction in cluster symmetry, limiting solid-state packing interactions between molecules and promoting solvation (Fig. 12).^126^

Likewise, the incorporation of aliphatic,^130^ pyridyl and amino^131^ functionalized TRIS ligands onto the POV–ethoxide scaffold was accomplished; however, only moderate improvements to cluster solubility was observed. Polyether-backed tripodal ligands vastly improved the solubility of this system, inspiring researchers to directly incorporate ether alcohols into the cluster framework and yielding V_6_O_7_(OR)_12_ (R = C_2_H_4_OCH_3_, C_2_H_4_OC_2_H_5_). Homoleptic ether–alkoxide bridged complexes showed promising solubility parameters (∼0.93 M); however, mixing with methoxide groups led to the generation V_6_O_7_(OC_2_H_4_OCH_3_)_12−*x*_(OCH_3_)_*x*_, which features the highest solubility of a POM-based complex in MeCN at 1.2 M.^127^

These studies have proven that direct surface functionalization of POVs enables tuneable solubility of the compound without interfering with core electronics. This provides both an atomically precise description of NC solubility, as ESI-MS and crystallographic characterization allows for determination of the hydrophobic ligand sphere, as well as informs design features, such as mixing both size and functionality of capping ligands to improve solvent compatibility of nanoparticles without impacting the electronic characteristics of the material.

### Ligand functionality dependence of POV–alkoxide electronic properties

Reducible metal oxides (RMOs) are applicable for a variety of chemical transformations due to their redox flexibility; gaining control over redox processes in these materials would allow for optimized activity. Where a particle's size has a significant effect on these properties, a NC's organic capping groups have also been touted as means by which one can tune a material's electronic properties following nanoparticle formation. For example, by adsorbing ligands with different electronic features, Rajh and co-workers were able to influence the reducing power of a TiO_2_ nanocrystalline electrode by more than 0.2 V as a consequence of inductively injected electron density from the capping group.^137^ In the same report, the authors found that the inclusion of carboxylates as capping moieties on the NC surface facilitates photoinduced charge separation, with the excited electrons localized in the conduction band of the metal oxide core, and holes being observed in the form of carboxyl radicals. In a subsequent study, the effect of capping groups on the band gap of several metal oxide materials (TiO_2_, ZnO_2_, and Fe_2_O_3_) was observed.^138^ It was found that both increased metal-capping group bond dipoles and electron donating character of the ligand decreased the band gap of the material, further demonstrating how careful modification of the surface of nanomaterials can have a profound impact on electronic structure of the core metal oxide.

Similar to those of RMOs, POMs have rich redox properties that can be modified by surface functionalization with organic “ligands”. The composition of these clusters allows for multi-electron reduction chemistry for these inorganic assemblies, which has prompted a great deal of research into the applications of these molecular assemblies in energy storage and catalysis. For in-depth analysis of the basis of the redox properties of POMs, we refer the reader to the recent review article by Ueda.^139^ Organic functional groups have been reported to exhibit significant control over the redox processes of tungstate and molybdate polyoxoanions. In particular, class II hybrid polyoxotungstate clusters (*i.e.* POMs which contain covalently-bound organic functional groups on the cluster surface) are able to facilitate redox processes by reducing the overall charge of the complex and structurally stabilizing reduced states. The full scope of modifications to the electrochemical properties of POMs have been recently reviewed by Kibler and Newton.^100^ Here, we will describe further advances in this space for POV clusters, namely the role bridging alkoxide ligands play in dictating the redox properties of iso- and mixed-valent vanadium oxide clusters.

Organic ligands bound to the surface of vanadium oxide clusters have been shown to tune the redox profile of the metal oxide assembly. In some of the earliest reports describing the synthesis and characterization of Lindqvist-type POV–alkoxide clusters, electrochemical analysis of the hexavanadate core reveals a strong dependence of the reduction potential of the cluster on the electronic properties of surface ligands. For example, Zubieta and co-workers describe a correlation between the electron-withdrawing/-donating properties of the functional group bound to the tertiary carbon of the TRIS capping ligand to the reduction potential of the first reduction event of the cluster (Table 2).^86^ Electron-withdrawing groups, such as terminal nitro, alcohol, and aryl substituents, inductively remove electron density from the Lindqvist core, resulting in an anodic shift of the first reduction process to more modest potentials. In contrast, electron-donating moieties, like alkyl or tertiary amino functionalities, add electron density to the vanadium oxide assembly, translating to increased energies required to inject additional electron density into the cluster core. Notably, a linear relationship between the cluster reduction potential and Hammet parameters of the pendant functionalities was observed, indicating that this site is a precise synthetic handle toward tuning cluster electronics.

**Table 2:** Reduction potentials of [V_6_O_13_(TRIS^R^)]^2−^ clusters as a consequence of the TRIS functional group

[V_6_O_13_(TRIS^R^)_2_]^2−^	Reduction potential (V) *vs.* Fc^0/+^
R = NO_2_	−0.67
R = CH_2_OH	−0.76
R = CH_2_(C_6_H_5_)	−0.85
R = CH_2_CH_3_	−1.17
R = CH_2_N(CH_3_)_2_	−1.18
R = CH_3_	−1.20

**Fig. 13:**
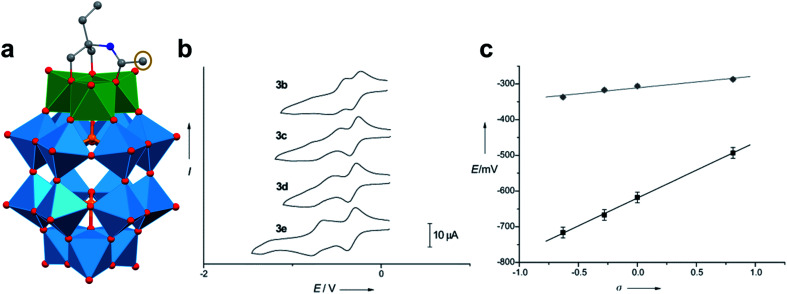
(a) Molecular structure of [P_2_V_3_W_15_O_59_{(OCH_2_)_2_C(C_2_H_5_)NHCOCH_3_}]^5−^ with electronically directing functionality circled in gold; colour code: V: green polyhedra, W: light blue polyhedra, O: red spheres, C: grey spheres, N: dark blue spheres. Hydrogen atoms, solvent molecules, and counterions omitted for clarity; (b) cyclic voltammograms of [P_2_V_3_W_15_O_59_{(OCH_2_)_2_C(C_2_H_5_)NHCOR}]^5−^ collected in acetonitrile, where R = C_6_H_4_-*p*-NO_2_ (3b), C_6_H_5_ (3c), C_6_H_4_-*p*-OCH_3_ (3d), C_6_H_4_-*p*-N(CH_3_)_2_ (3e); and (c) plot of V-based reduction potentials *vs.* Hammet parameters (*σ*) of R groups. This figure has been adapted/reproduced from ref. 140 with permission from the Wiley, copyright 2009.

A similar effect was reported by Hasenknopf and co-workers on the organofunctionalized vanadium-containing Wells-Dawson cluster, [P_2_V_3_W_15_O_59_{(OCH_2_)_2_C(Et)NHCOCH_3_}]^5−^ (Fig. 13). The organofunctionalized assembly was synthesized *via* exchange of bridging oxide ligands at the vanadate sites of the parent heterometallic polyoxoanion, [P_2_V_3_W_15_O_62_]^9−^, for 2-arylamino-2ethyl-1,3-propanediol derivatives. The ligand is rendered tridentate by coordination of the amide-carbonyl oxygen atom to two vanadium atoms, effectively inserting this functional group into the electronic structure of the vanadium oxide moieties of this POM (Fig. 13a).^140^ Organofunctionalization results in enhanced vanadium ion communication, evidenced by splitting of the single V-based redox wave of [P_2_V_3_W_15_O_62_]^9−^ into a multi-step redox wave (Fig. 13b).^140,141^*Para*-functionalization of the aromatic substituents bound to the carbonyl of the amide, accomplished prior to cluster incorporation, provided a direct means for probing the influence of electron donation to the POM's electrochemical processes.

Again, electron withdrawing functional groups produced lower relative reduction potentials *versus* substituents with electron donating properties, resulting in a direct relationship between reduction potential and the functional groups’ apostrophe Hammet parameters (Fig. 13c). In the case of this mixed-metal POM cluster, the vanadate ions bound directly to the conjugated organic moiety are highly adjustable sites for accessing unique properties. Collectively, these studies indicate that the redox processes of the metal oxide core can be tuned through judicious choice of functional groups on the organic scaffold. This results in predictable control over cluster electronics *via* atomically precise modifications to the surface of the vanadium oxide assembly.

Another family of POV–alkoxide clusters with extensively characterized electrochemical properties are the Lindqvist-type organofunctionalized hexavanadate assemblies featuring twelve bridging alkoxide functional groups, [V_6_O_7_(OR)_12_]. As described above, these clusters entirely lack bridging oxide ligands (*i.e.* all bridging positions are occupied by alkoxide moieties) and are isolated in low-valent oxidation states (*e.g.* the singly-anionic cluster isolated directly from solvothermal synthesis possesses an electronic distribution of vanadium oxidation states of V^IV^_5_V^V^). In contrast to the modest electrochemical profile of the TRIS-capped, high-valent (V^V^_6_) Lindqvist ions reported by Zubieta which feature a single reduction event, the reduced variants of these POV–alkoxide assemblies possess four quasi-reversible redox events spanning ∼2 V. Their rich redox properties have resulted in these clusters being a focus of energy storage application-driven research.^125–127,130,136,142^

**Fig. 14:**
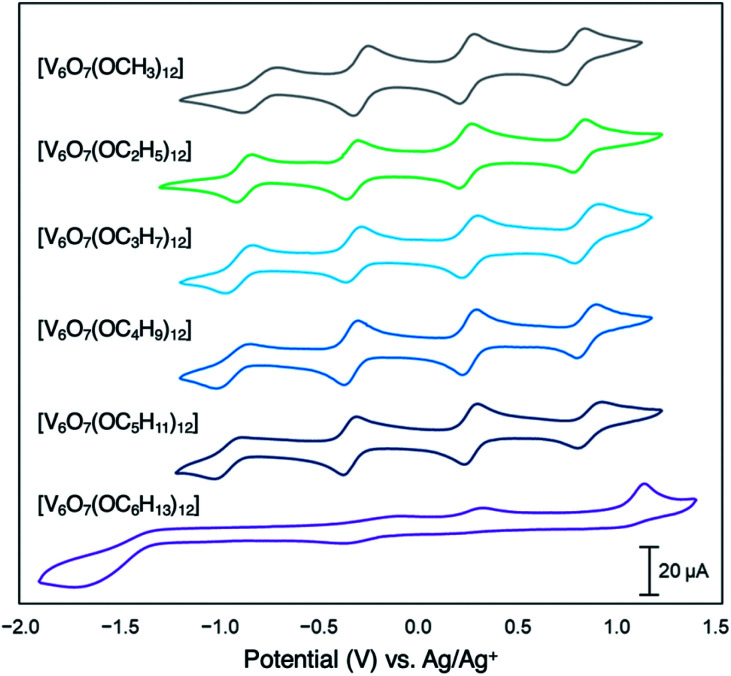
Cyclic voltammograms of homoleptic POV–alkoxide clusters at 1 mM concentration measured in acetonitrile with 0.1 M [^*n*^Bu_4_]PF_6_ supporting electrolyte. Scan rate 100 mV s^−1^, open circuit potential ∼0 V for all complexes. This figure has been adapted/reproduced from ref. 126 with permission from the Royal Society of Chemistry, copyright 2019.

Since their original synthesis in 2003 by Hartl, Matson has extended these studies to a series of POV–alkoxide clusters featuring alkoxide functional groups of varying lengths (*vide supra*). Notably, the CVs of all these POV–alkoxide clusters are nearly identical, with minimal observable shifts in redox potentials across the series (Fig. 14,Table 3).^126^ Discrepancies in the electronic properties of these clusters can be observed by analysis of clusters' redox stability; while attempts to oxidize the POV–methoxide cluster to its di-cationic form, [V_6_O_7_(OCH_3_)_12_]^2+^, manifests in cluster degradation, POV clusters supported by alkoxide ligands with carbon chains ranging from C_2_H_5_ to C_5_H_11_ inductively stabilize the vanadate core at higher potentials, resulting in an oxidatively robust Lindqvist assembly.

**Table 3:** Electrochemical parameters of [V_6_O_7_(OR)_12_] clusters^a^

Complex	V^IV^/V^IV^_5_V^V^ couple	V^IV^_5_V^V^/V^IV^_4_V^V^_2_ couple	V^IV^_4_V^V^_2_/V^IV^_3_V^V^_3_ couple	V^IV^_3_V^V^_3_/V^IV^_2_V^V^_4_ couple	Ref.
[V_6_O_7_(OCH_3_)_12_]	−0.72 (0.99)	−0.22 (1.01)	0.30 (1.00)	0.85 (1.02)	136
[V_6_O_7_(OC_2_H_5_)_12_]	−0.88 (0.98)	−0.34 (1.00)	0.22 (1.00)	0.79 (1.01)	136
[V_6_O_7_(OC_3_H_7_)_12_]	−0.89 (0.98)	−0.33 (0.99)	0.25 (1.02)	0.83 (1.03)	125
[V_6_O_7_(OC_4_H_9_)_12_]	−0.94 (0.96)	−0.35 (0.99)	0.24 (1.00)	0.83 (1.03)	125
[V_6_O_7_(OC_5_H_11_)_12_]	−0.96 (0.89)	−0.36 (0.98)	0.25 (1.06)	0.85 (1.07)	126
[V_6_O_7_(OC_2_H_4_OCH_3_)_12_]	−0.74 (1.09)	−0.16 (1.01)	0.34 (0.98)	1.06 (0.22)	127
[V_6_O_7_(OC_2_H_4_OC_2_H_5_)_12_]	−0.70 (1.18)	−0.17 (1.03)	0.34 (1.00)	1.15 (0.14)	127
[V_6_O_7_(OC_2_H_5_)_12−*x*_(OCH_3_)_*x*_]	−0.85 (1.05)	−0.32 (1.04)	0.24 (1.01)	0.81 (1.03)	126
[V_6_O_7_(OC_3_H_7_)_12−*x*_(OCH_3_)_*x*_]	−0.83 (0.89)	−0.30 (1.05)	0.27 (1.01)	0.83 (1.01)	126
[V_6_O_7_(OC_4_H_9_)_12−*x*_(OCH_3_)_*x*_]	−0.84 (0.95)	−0.39 (0.99)	0.28 (1.03)	0.83 (1.04)	126
[V_6_O_7_(OC_5_H_11_)_12−*x*_(OCH_3_)_*x*_]	−0.84 (0.92)	−0.29 (1.04)	0.27 (1.06)	0.94 (1.08)	126
[V_6_O_7_(OC_2_H_4_OCH_3_)_12−*x*_(OCH_3_)_*x*_]	−0.67 (1.12)	−0.17 (1.04)	0.31 (1.09)	0.82 (0.32)	127
[V_6_O_7_(OC_2_H_4_OC_2_H_5_)_12−*x*_(OCH_3_)_*x*_]	−0.67 (1.10)	−0.19 (0.95)	0.31 (0.98)	0.85 (0.87)	127

a Standard potentials (measured *vs.* Ag/Ag^+^) identified using cyclic voltammetry at 100 mV s^−1^ of 1 mM solutions of each complex with 0.1 M [^*n*^Bu_4_N]PF_6_ supporting electrolyte in acetonitrile. Values in parentheses indicate ratios of the cathodic to anodic peak heights (*i*_c_/*i*_a_).

The general lack of influence of the alkoxide identity on this cluster family's electronics is striking, considering that subtle modifications to the cluster core manifest in substantial changes in the redox profile of the system. For example, substitution of a single alkoxide ligand in the Lindqvist assembly for a bridging oxide moiety translates to substantial shifts in the redox potentials for the resultant POV–alkoxide clusters (*E*_1/2_([V_6_O_8_(OCH_3_)_11_]) = −0.73, −0.19, +0.36, +0.94 measured in MeCN *vs.* Fc^+/0^; *E*_1/2_([V_6_O_7_(OCH_3_)_12_]) = −0.28, +0.25, +0.79 measured in MeCN *vs.* Fc^+/0^).^46^ This result provides important insight for the materials science community; atomic precision in the case of population density of surface ligands has a dramatic effect on the redox properties of the cluster core.

In an effort to realize the full extent of electronic communication between alkoxide ligands and the hexavanadate core in mixed-valent POV–alkoxide clusters, Matson and co-workers reported the synthesis of a library of vanadium oxide assemblies featuring TRIS functional groups that resemble those originally reported by Zubieta and co-workers (Fig. 11b).^130,131^ Electron-withdrawing (*e.g.* NO_2_, C_5_H_4_N) and electron-donating (*e.g.* CH_3_, C_2_H_5_) functional groups were appended to the surface of the cluster at the tertiary carbon; interrogation of the redox properties of the cluster core *via* CV revealed minimal changes in the potential of the redox events for all assemblies (∼0.10 V). This result is in stark contrast to that observed with the previously described, high-valent Lindqvist ions, [V_6_O_13_(TRIS^R^)_2_]^2−^ (R = CH_3_, C_2_H_5_, NO_2_). The authors conclude that the limited influence of ligand identity on the electrochemical profile of the mixed-valent POV–alkoxide is due to saturation of the cluster surface by alkoxide ligands, stabilizing the redox properties of the vanadate core and decoupling the TRIS ligand electronics from that of the cluster.

Taking advantage of electronic insulation imparted by alkoxide saturation, Spandl showed that the electrochemical profiles of their post-synthetically modified, TRIS-functionalized Lindqvist POV–alkoxides remain completely unaffected, even after covalently linking multiple clusters to one another.^128^ CV of the generated trimeric cluster complexes revealed a largely unchanged redox profile to the monomeric complex, with redox potentials conserved between the mono and trimeric systems (Δ*E*_1/2_ < 0.1 V). Therefore, tethering the POVs allowed for the generation of a multi-cluster scaffold without interfering with the bound clusters' electron transfer characteristics, an approach to material design that allows for the build-up of complex, functional materials with desired characteristics from its molecular precursors.

It has also been shown that the incorporation of sterically incumbering ligands may hinder heterogeneous electron transfer altogether. Nanocrystalline materials, which are typically formed using long-chain surfactants such as oleic acid, are structurally stable with this capping group; however, these systems can suffer from insulating effects which inhibit interparticle conductivity. It has been shown that post-synthetic exchange of capping ligands for smaller moieties, such as pyridine, allows for improved interparticle conductivity; however, these capping groups decrease particle stability, resulting in aggregation to form bulk materials.^143^ Therefore, understanding the limits to conductivity and stability is highly important toward designing and optimizing robust electroactive materials.

Matson and co-workers reported this effect with a POV–alkoxide cluster derived from *n*-hexanol, resulting in formation of the hexavanadate assembly, V_6_O_7_(OC_6_H_13_)_12_. The bulky, nonpolar surface ligands shield the cluster core from a glassy carbon electrode, hindering electron transfer events (Fig. 14).^126^ Notably, the *n*-pentanol derivative of this compound was shown to exhibit the characteristic redox profile of this class of complexes (four quasi-reversible redox processes spanning ∼2 V), indicating that a threshold of ligand shielding for heterogeneous charge transfer processes in these systems exists.

By using atomically precise POV–alkoxides to carefully modify and observe the properties that capping ligands impart on these systems, researchers are able to reach an atomistic description of the properties observed with organic-capped nanoparticles, such as surface ligand density, solvent compatibility, substrate attraction, and charge transfer. Not only does this provide powerful fundamental understanding of metal oxides, it allows for a molecular-scale means for developing new capping groups to suit specific aims. Indeed, these studies could allow materials scientists to precisely modify desired properties prior to ligand incorporation into nanocrystalline architectures, which are more challenging to study with high sensitivity.

## Atomically “doped” polyoxovanadate clusters

The widespread use of RMOs in heterogenous catalysis is credited to the extensive variety of atomic compositions and surface structures of these materials.^1,19,144–146^ Indeed, this versatility results in a broad range of chemical and physical properties, making metal oxides attractive for transformations spanning various fields.^145,147–150^ While there are several routes used to modulate the properties of extended solids, the most prominent method involves using cationic or anionic dopants.^13,144,151,152^ “Doping” of these materials can be defined as the introduction of trace heteroatom impurities to the lattice of a semiconducting solid, which results in an alteration of the material's properties. Experimental and computational investigations into the consequences of dopant addition has revealed that heteroion incorporation could result in both structural (*i.e.* vacancy generation, lattice distortions) and electronic (*i.e.* conductivity, electron delocalization, carrier density) modifications to the extended solid. However, difficulties in experimentally monitoring *in situ* catalytic transformations has made an understanding of the role of metal or nonmetal dopants largely speculative.^17,18,153–155^ As such, metal oxide clusters, such as polyoxometalates, can be used to model bulk materials, providing fundamental information surrounding the influence of these impurity ions on the properties of heterogenous catalysts.

### Cationic dopants in molecular vanadium oxide assemblies

**Fig. 15:**
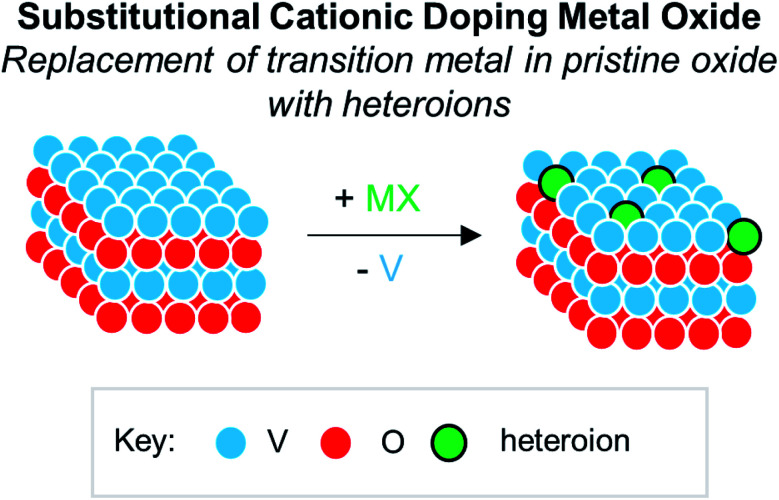
Substitutional cationic doping of extended solids of V_*x*_O_*y*_; a vanadium centre that makes up the pristine lattice is replaced with a heteroion.

Numerous reports have demonstrated that the introduction of cationic dopants, with either heterometal (*e.g.* d- or f-block) or nonmetal elements (*e.g.* s- or p-block), can significantly alter the physical, chemical, electronic, and optical properties of an oxide surface.^13,144^ In particular, substitutional cationic doping involves the exchange of some fraction of metal ions that initially compose the pristine metal oxide with a cationic heteroion (*i.e.* M_*x*_O_*y*_ + D → D_*a*_M_*x*−*b*_O_*y*−*c*_) (Fig. 15). Theoretical investigations have revealed that perturbations invoked due to substitutional cationic doping is directly related to the metal valencies of the dopant and the metal oxide.^13,156^ For example, if the dopant has the same valence state as the initial metal ions, generally, only the RMO size and element–oxygen bond strength (either M–O or D–O) is altered. In contrast, when the dopant has a different oxidation state to that of metal ions within the pristine material, a charge imbalance occurs and results in more severe structural and electronic perturbations. While the consequences of substitutional doping have been thoroughly investigated, challenges still remain in obtaining a complete understanding of the influence of these defects on the properties of RMOs. The inability to establish a precise thermodynamic and/or kinetic control over dopant incorporation levels, and the subsequent difficulties of analysing doped materials with varying concentrations of heteroions, makes the correlation between dopant addition and the change in metal oxide properties challenging.^157–159^ Given this fact, the use of discreet, cationically doped polynuclear molecules as atomically precise models for bulk materials can provide crucial information into structural and electronic consequences of substitutional doping.

### Synthesis and structure of “doped” POV clusters

**Fig. 16:**
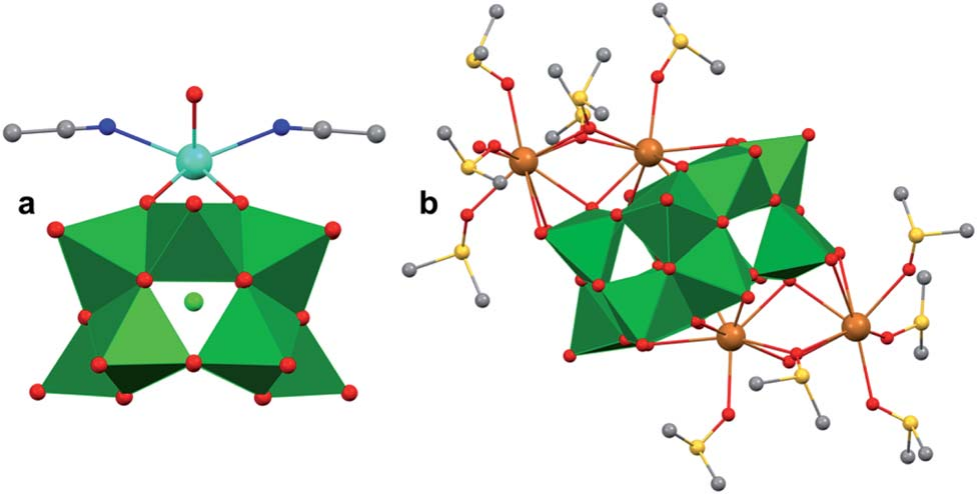
Molecular structures of (a) [GdV_12_O_32_Cl]^1−^ and (b) [Ba_4_V_14_O_38_(NO_3_)]. Colour code: V: green polyhedral, Gd: cyan, Ba: brown, O: red, N: blue, C: grey, Cl: light green. S: yellow spheres. Hydrogen atoms, solvent molecules and counter cations are omitted for clarity.

The polynuclear composition and delocalized electronic structure of polyoxometalates makes them excellent model systems for bulk metal oxides.^22,160^ With the overarching goal of analysing the effects of introducing heterometals onto the surface of M_*x*_O_*y*_ using heteropolyoxoanions as molecular models, it is important to consider the analogous types of heteroion-doped cluster complexes. Various types of heterometal-installed POMs have been synthesized through the addition of transition metal salts to Lacunary-type clusters, which are composed of unsaturated sites, or ‘vacant’ pockets, that can accommodate heteroions.^52,161–163^ While the assembly of these multi-metallic POMs, *via* Lacunary starting materials, is well-studied for molybdenum and tungsten clusters, considerably less research has focused on the isolation of the heteropolyoxovanadate congeners. This is mainly due to the small ionic radius of vanadium which presents challenges in isolating stable Keggin- and Dawson-type POVs, as well as their corresponding Lacunary derivatives.^51^ Seminal contributions into the generation of heteroion-installed polyoxovanadates have been led by Streb^164–174^ and others^34,175–177^ who have developed synthetic methods toward coordinating various alkali ions, transition metals, or lanthanides to vanadium oxide clusters (*i.e.* [V_12_O_32_Cl]^*n*^ → [M_*x*_V_12_O_32_Cl]^*n*^: *x* = 1, 2; M = Mn^II^, Fe^III^, Co^II^, Cu^II^, Zn^II^, Ce^III^, Gd^II^; Fig. 16). However, in analogy to doped metal oxide materials, isolation of these types of heterometallic clusters would most closely resemble cation chemisorption onto RMO surfaces. As such, these types of polynuclear assemblies will not be discussed in this review, however the reader is encouraged to read review articles by P. Kögerler,^52^ M. Nyman and P. C. Burns^163^ for a more expansive overview of these types of heterometal-coordinated POVs.

The first report of substitutionally-doped, heterometal-functionalized polyoxovanadates were published by Pope and Flynn in 1971, who described methods toward generating mixed vanadyl–tungsten Lindqvist clusters where vanadyl ions were replaced by tungsten–oxo moieties, [V_6−*x*_W_*x*_O_19_]^*n*−^.^178^ The authors found that heating mixtures of vanadium and tungsten salts, or directly using the metal oxides (*i.e.* KVO_3_, K_2_WO_4_, Na_4_V_2_O_7_, WO_3_, V_2_O_5_), resulted in the formation of [VW_5_O_19_]^*n*−^ or [V_2_W_4_O_19_]^*n*−^. Since this initial report, Streb and co-workers have disclosed synthetic methods for generating the analogous vanadium–molybdenum clusters.^179,180^

In comparing the heterometallic clusters to that of [M_6_O_19_]^*n*−^ (M = Mo, W), the authors found that heterometal incorporation can be used to modify the properties of POMs, in particular, resulting in visible light absorption and subsequent photocatalytic activity of [V_*x*_M_6−*x*_O_19_]^*n*−^: the incorporation of two vanadium ions within the multimetallic POV assembly (*e.g.* [V_2_M_4_O_19_]^*n*−^; M = Mo, W) resulted in higher quantum efficiencies for the photooxidation of organic substrates.^180^ Density functional theory calculations of the heterometallic POVs show that this enhanced reactivity is a result of low-energy ligand to metal charge transfer from the bridging oxo moieties of the POV to empty vanadium d-orbitals.^179^

**Fig. 17:**
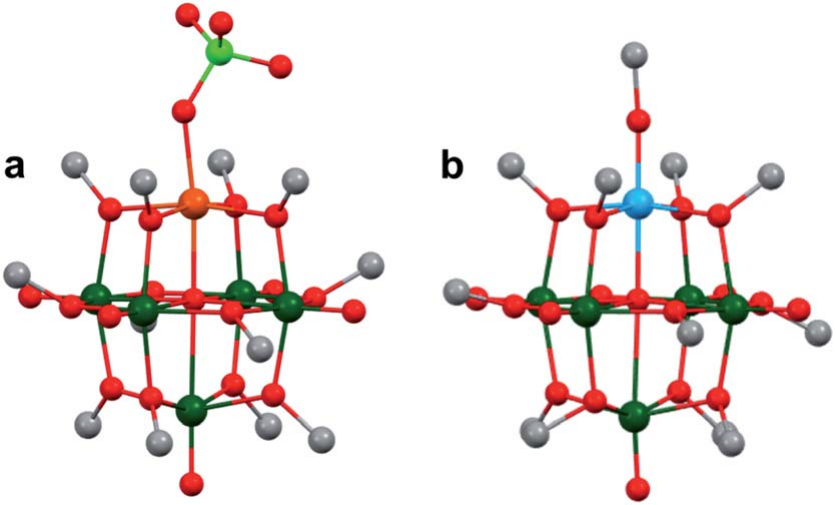
Molecular structures of (a) [V_5_O_6_(OCH_3_)_12_Fe(ClO_4_)] and (b) [V_5_O_6_(OCH_3_)_12_Ti(OCH_3_)]^1−^. Colour code: V: dark green, Fe: orange, Ti: blue, O: red, C: grey, Cl: green. Hydrogen atoms, solvent molecules and counter cations are omitted for clarity.

More recently, Matson and co-workers have investigated into the consequences of heterometal installation with alkoxide-bound Lindqvist polyoxovanadate (POV–alkoxide) clusters, [V_6_O_7_(OCH_3_)_12_]^*n*^ (Fig. 16). Through solvothermal, self-assembly protocols, Li *et. al.* reported the synthesis of an iron-functionalized POV–alkoxide cluster, [V_5_O_6_(OCH_3_)_12_FeX] (X = Br, SO_3_CF_3_, OClO_3_) (Fig. 17a), where the heterometallic iron centre replaces a terminal vanadyl ion and results in the incorporation of Fe^III^ directly within the metal oxide core.^181,182^ Indeed, growth of single crystals suitable for X-ray analysis confirmed that the FeX moiety is directly bound to the central μ^6^–oxo centre (O_c_), with a Fe–O_c_ bond length of 2.211(7) Å, slightly truncated from that of the hexavanadium derivative (*e.g.* [V_6_O_7_(OCH_3_)_12_]^1−^; V–O_c_ (avg) 2.31 Å; Table 4). These results revealed that introduction of a d^5^ metal ion into the POV–alkoxide cluster results in structural perturbation of the vanadium oxide core; the relatively electron-rich iron ion is pulled toward the centre of the vanadium oxide cluster. Following the synthesis and characterization of the redox-series of iron-installed POV–alkoxide clusters, Matson and co-workers disclosed synthetic methods toward isolating the analogous POVs with a titanium (Fig. 17b), hafnium, zirconium,^183^ or gallium^184^ heteroion. Each cluster was isolated *via* solvothermal methods; a vanadium(v) alkoxide, VO(OR)_3_, was added to the corresponding heteroion precursor (*i.e.* GaCl_3_, Ti(OCH_3_)_4_, M(O^*t*^Bu)_4_; M = Hf, Zr) in the presence of a reductant, resulting in the formation of the desired substitutionally-doped POV–alkoxide clusters. In all cases, analysis *via* single crystal X-ray diffraction confirmed that introduction of heterometals into the reaction mixtures resulted in the assembly of a alkoxovanadium cluster featuring a M–X (*e.g.* Ga–Cl, M–OCH_3_; M = Ti, Hf, Zr) moiety with *pseudo-O*_h_ geometry. Similar to that of the initial iron-installed cluster, the M–O_c_ bond length for each multimetallic cluster was decreased in comparison to that of the homometallic congener (Table 4).

**Table 4:** M–O_c_ bond lengths of parent hexavanadate and heterometal-installed POV–alkoxide clusters

POV–alkoxide cluster	Ox. state distribution	M–O_c_ (Å)
[V_6_O_7_(OCH_3_)_12_]^0^	V^IV^_5_V^V^	2.31(avg.)
[V_5_O_6_(OCH_3_)_12_(FeCl)]^0^	V^IV^_3_V^V^_2_Fe^III^	2.2428(8)
[V_5_O_6_(OCH_3_)_12_(GaCl)]^0^	V^IV^_3_V^V^_2_Ga^III^	2.225(11)
[V_5_O_6_(OCH_3_)_12_(TiOCH_3_)]^1−^	V^IV^_5_Ti^IV^	2.042(5)
[V_5_O_6_(OCH_3_)_12_(TiOCH_3_)_2_]^0^	V^IV^_5_Ti^IV^_2_	2.1183(13), 2.1336(13)
[V_5_O_6_(OCH_3_)_12_(ZrOCH_3_)]^1−^	V^IV^_5_Zr^IV^	2.153(2)
[V_5_O_6_(OCH_3_)_12_(HfOCH_3_)]^1−^	V^IV^_5_Hf^IV^	2.147(5)

Whereas all previous reports of these Lindqvist alkoxovanadium clusters featured the installation of a single transition metal centre, Matson and co-workers have also discovered a method for isolating a dititanium-functionalized POV–alkoxide cluster by simply increasing the amount of the titanium alkoxide precursor and reaction time.^185^ Notably, the structure forms exclusively a single product, with the two titanium centres located *cis* to one another. Interestingly, the introduction of these *cis*-titanium centres results in similar structural perturbations to that of the mono-titanium cluster; shortened Ti–O_c_ bonds and elongated V–O_c_ bond lengths were observed (Table 4).

While the POV–alkoxide clusters show structural changes upon heterometal incorporation, these perturbations are limited to alteration in M–O_c_ or V–O_c_ bond lengths and angles. In contrast, theoretical investigations into consequences of the installation of substitutional dopants in bulk vanadium oxide materials have revealed that cationic doping can result in dramatic structural and electronic changes that substantially influence of properties of the material.^5,13^ For example, replacement of V^5+^ ions within V_2_O_5_ with cations in the M^2+^ and M^3+^ oxidation states can result in the generation of oxygen–atom vacancies as a method for charge-compensation. Additional consequences, such as modification of stability, size or morphology, shift of the band gap energy, and increased conductivity have also been observed.^186–188^

### Electronic consequences of cationic dopants in POVs

The redox properties of polyoxometalates have been well established to be dependent on the molecular composition and structure of the polynuclear assembly.^46,189^ As such, electrochemical analysis of these heterometallic POV–alkoxides *via* CV can provide valuable information including (1) the electron delocalization between transition metal centres that compose the POM, (2) the reversibility of the electron transfer processes, and (3) provide knowledge of the oxidizing or reducing power of the cluster *via* the location of the redox events. Collectively, these studies present a method toward understanding the electronic influence of heterometal dopants on POV clusters. As such, the Matson laboratory analysed the electrochemical profile of each multimetallic cluster to interrogate the electronic consequences of substitutional doping in these multimetallic assemblies.

**Fig. 18:**
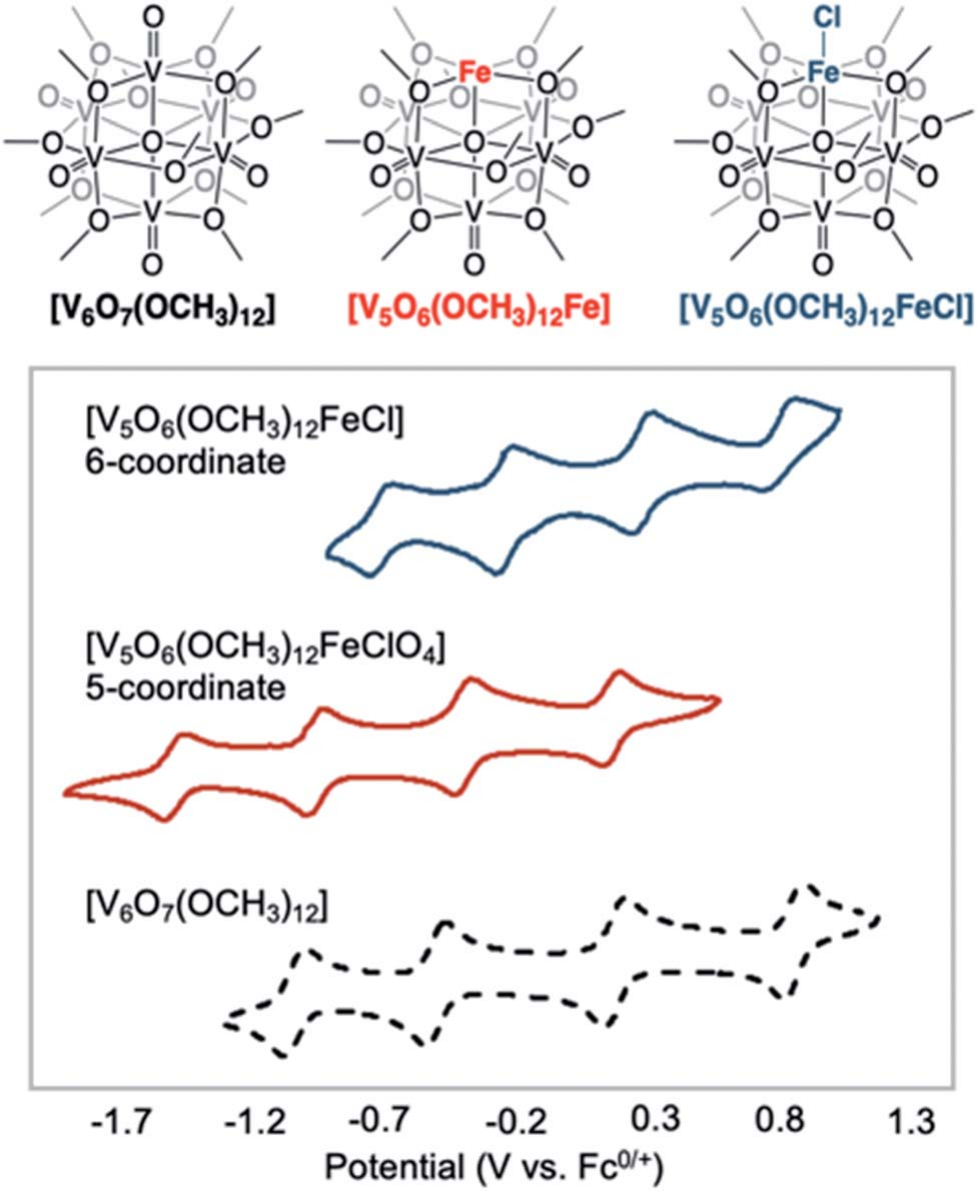
Cyclic voltammograms of parent POV–alkoxide clusters and iron-functionalized POV–alkoxide clusters recorded in acetonitrile with 0.1 M [^*n*^Bu_4_N]PF_6_ as the supporting electrolyte. This figure has been adapted/reproduced from ref. 190 with permission from Wiley, copyright 2020.

Fig. 18 compares the CV of the parent, homometallic cluster, [V_6_O_7_(OCH_3_)_12_]^*n*^ to the electrochemical profiles of iron-functionalized POV–alkoxide clusters. Notably, the introduction of iron into the hexavanadate scaffold did not change the number of redox events (Table 5).^181,182,184^ The CV of complex [V_5_O_6_(OCH_3_)_12_Fe(ClO_4_)] is cathodically shifted in comparison to the parent all-vanadium assembly, extensive characterization of the redox-series of FePOV–alkoxide clusters revealed that the four redox events observed in the CV correspond to V^IV^/V^V^ redox couples; Mössbauer and X-ray absorption spectroscopy further verified that the iron centre retains its 3+ oxidation state across all charge states.^182^

**Table 5:** Electrochemical parameters of parent hexavanadate and iron-installed POV–alkoxide clusters in MeCN(v) *vs*. Fc^0/+^

Redox couple of [V_6_O_7_(OCH_3_)_12_]	*E*_1/2_	Redox couple of [V_5_O_6_(OCH_3_)_12_FeCl] (6-coordinate)	*E*_1/2_	Redox couple of [V_5_O_6_(OCH_3_)_12_Fe] (5-coordinate)	*E*_1/2_
[V^IV^_6_]/[V^IV^_5_V^V^]	−1.01	[V^IV^_5_Fe^III^]/[V^IV^_4_V^V^Fe^III^]	−0.68	[V^III^V^IV^_4_Fe^III^]/[V^IV^_5_Fe^III^]	−1.46
[V^IV^_5_V^V^]/[V^IV^_4_V^V^_2_]	−0.49	[V^IV^_4_V^V^Fe^III^]/[V^IV^_3_V^V^_2_Fe^III^]	−0.21	[V^IV^_5_Fe^III^]/[V^IV^_4_V^V^Fe^III^]	−0.94
[V^IV^_4_V^V^_2_]/[V^IV^_3_V^V^_3_]	+0.21	[V^IV^_3_V^V^_2_Fe^III^]/[V^IV^_2_V^V^_3_Fe^III^]	+0.31	[V^IV^_4_V^V^Fe^III^]/[V^IV^_3_V^V^_2_Fe^III^]	−0.36
[V^IV^_3_V^V^_3_]/[V^IV^_2_V^V^_4_]	+0.90	[V^IV^_2_V^V^_3_Fe^III^] [V^IV^V^V^_4_Fe^III^]	+0.83	[V^IV^_3_V^V^_2_Fe^III^]/[V^IV^_2_V^V^_3_Fe^III^]	+0.21

In a subsequent publication, Matson and co-workers investigated the consequence of coordination number (*e.g.* five- *vs.* six-coordinate Fe^III^) on the electrochemical profile of the iron-functionalized POV–alkoxide clusters (Fig. 18,Table 5).^190^ While not changing the oxidation state of the metal centre, modifying the coordination sphere of iron manifested in drastic changes to the redox potentials of the four electrochemical processes of the Lindqvist core. When the Fe centre within the POV is ligated to an X-type ligand (*i.e.* 6-coordinate; V_5_FeCl or V_5_FeOCN), the 4-step redox wave is only slightly shifted from that of the homometallic congener. However, upon investigating the redox profile of the coordinatively unsaturated cluster (*i.e.* 5-coordinate, with only a weakly coordinated ClO_4_^1−^ anion) the redox events were shifted anodically by ∼0.7 V. Density functional theory analysis of the electronic structures of both the five- and six-coordinate compounds revealed that modification of the geometry of the iron centre (*i.e.* 5-coordinate, square pyramidal; 6-coordinate, octahedral) results in significant changes in the highest occupied molecular orbital energies of the clusters. For example, the first and second oxidative events were calculated to be more accessible for the iron-chloride installed cluster in comparison to that of the five-coordinate cluster. This change in electronic structure upon ligation of an X-type ligand to the heteroion manifests in increased oxidative stability, ultimately translating to the observed shift in redox potential of the four vanadium-based electrochemical processes.

**Fig. 19:**
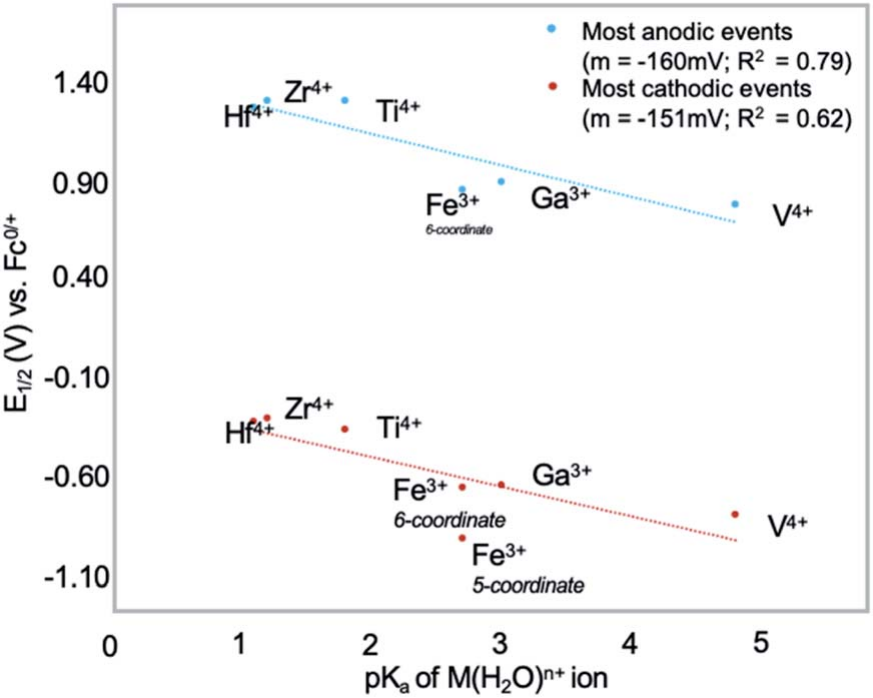
Relationship between the Lewis acidity of the heterometal of heteroions (p*k*_a_ (p*k*_a_(H_2_O))) and the *E*_1/2_ values of the Lindqvist POV–alkoxides. Plots feature most cathodic (V^IV^_5_M/V^IV^_4_V^V^M) and most anodic (V^IV^_2_V^V^_3_M/V^IV^V^V^_4_M) redox couples (M = Hf^4+^, Zr^4+^, Ti^4+^, Fe^3+^, Ga^3+^, V^4+^) in MeCN. This figure has been adapted/reproduced from ref. 190 with permission from Wiley, copyright 2020.

Similar anodic shifts of the electrochemical processes were observed in the case of POV–alkoxides functionalized with group 4 (M = Ti, Hf, Zr) transition metal ions (*i.e.* V_5_O_6_(OCH_3_)_12_(MOCH_3_)).^183^ The electrochemical characterization of these heterometallic POVs permitted Meyer *et al.* to establish a model to predict electronic perturbations of the metal oxide cluster upon cationic substitutional doping.^184,190^ The authors analysed the relationship between the Lewis acidity of the heterometal (*e.g. via* the aqueous p*k*_a_ (p*k*_a_(H_2_O)) of heteroions) and the *E*_1/2_ values of the Lindqvist metal oxides, finding a positive correlation (*R*^2^ = 0.79–0.90) (Fig. 19). Upon investigating these relationships for each redox event of the heterometallic clusters, the authors found significant differences between the slope (*i.e. E*_1/2_/p*k*_a_) for the most anodic (−160 mV/p*k*_a_) and cathodic (−143 mV/p*k*_a_) redox events, suggesting that the Lewis acidity strongly influences the redox properties clusters in their most-oxidized charged-states.

In contrast to the general changes observed upon substitutionally doping POV–alkoxide clusters, the titanium-functionalized Lindqvist cluster features unique electronic and physical properties.^189^ While the electrochemical profile of [V_6−*x*_O_6_(OCH_3_)_12_(TiOCH_3_)_*x*_]^*n*^ (*x* = 1, 2) is similar to that of the other heterometal-installed POV–alkoxide clusters, featuring four quasi-reversible V^IV^/V^V^ redox events, expanding the electrochemical window of the mono- and dititanium-functionalized clusters to more reducing potentials revealed additional events which were attributed to Ti^IV/III^ redox chemistry (*e.g.* TiV_5_: −2.07 V; Ti_2_V_4_: −2.10, −1.58 V). These results are essential for understanding the role of introducing cationic dopants to POVs, revealing that not only can the voltage window be significantly influenced but also adding in redox-active metal ions to these polynuclear assemblies can increase the number of reducing equivalents stored within the metal oxide cluster (*i.e.*V_6_O_7_: 4e^−^; TiV_5_: 5e^−^; Ti_2_V_4_: 6e^−^).

### Anionic dopants in molecular vanadium oxide assemblies

**Fig. 20:**
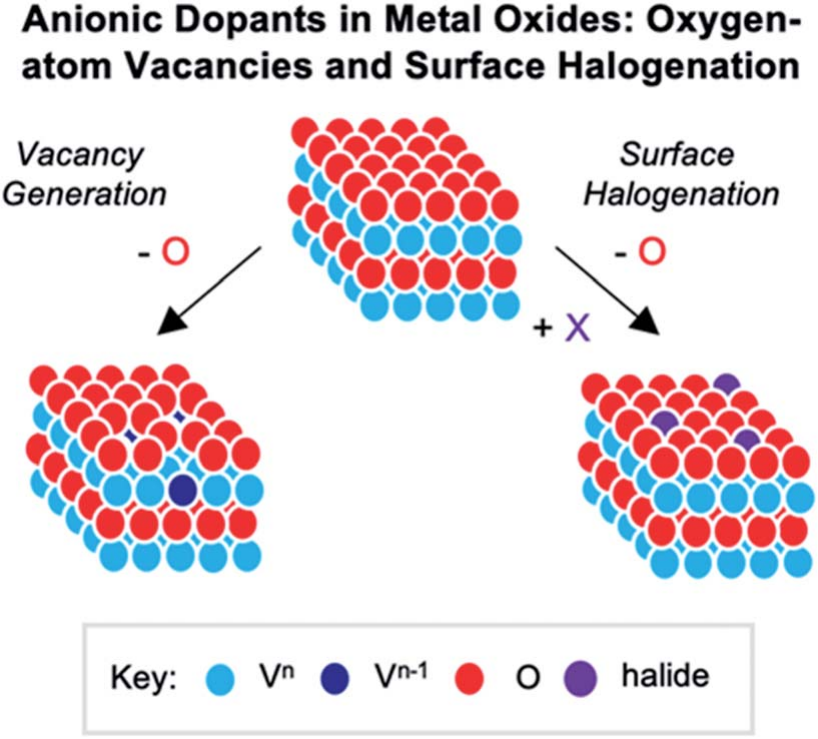
Cartoon depiction of anionic defects in metal oxides.

There are two main methods used to introduce anionic dopants into vanadium oxides (Fig. 20). The most prominent method involves the removal of lattice oxygen atoms to form oxygen-vacant sites on the surface of the material.^14,15,191–193^ An alternative method to anionically-doping vanadium oxide materials is through the substitution of oxide moieties for non-oxygen anions such as halides,^194–200^ sulfides,^201–203^ or nitrides.^204,205^ Experimental and computational investigations have shown that both physical and chemical properties are significantly influenced by the introduction of these anionic dopants. Indeed, numerous studies have revealed that increasing the concentration of oxygen-deficient vanadium centres in V_*x*_O_*y*_ greatly enhances the catalytic activity of these materials toward small molecule activation processes, as these vacant sites are proposed to be the active sites of catalysis.^7,8,144^

Additionally, other physicochemical properties of doped-vanadium oxides, such as the metal-to-insulator phase transition temperature, and band edge energies are significantly altered upon addition of non-oxygen anions in comparison to the parent V_*x*_O_*y*_ material.^194–200^ While the changes observed upon anionically-doping vanadium oxides are likely a direct result of V=O bond cleavage or V–E (E = Cl^1−^, Br^1−^, F^1−^, N^3−^, S^2−^) bond formation, difficulties in *in operando* characterization has limited our understanding of the precise role of these anionic dopants in catalysis, to date. Thus, to overcome these challenges, the synthesis and characterization of molecular polyoxovanadate clusters, bearing oxygen atom defects and/or non-oxygen substituents, provides an avenue for investigating the influence of anionic dopants on the properties of bulk vanadium oxide materials.

### Atomically precise models of O-atom vacancies in RMOs

In 2018, the Matson laboratory reported the first example of oxygen atom abstraction from a Lindqvist polyoxovanadate assembly.^206^ Upon addition of V(Mes)_3_ (Mes = 2,4,6-trimethylbenzene) to the POV–alkoxide cluster, [V_6_O_7_(OCH_3_)_12_]^1−^ (ox. state distrib.: V^IV^_5_V^V^), the authors noted oxygen atom transfer from the cluster to the vanadium alkyl complex, resulting in the formation of an ‘oxygen-atom vacancy’. Characterization of the oxygen-deficient cluster, [V_6_O_6_(OCH_3_)_12_]^1−^, revealed that O-atom abstraction results in reduction of the single V^V^=O moiety embedded within the anionic parent cluster to a V^III^ ion (ox. state distrib: V^III^V^IV^_5_). Since this seminal report, additional investigations into V=O bond cleavage from alkoxovanadium clusters have revealed that tertiary phosphanes^207^ and organic acids^208^ can facilitate analogous post-synthetic modifications on POV–alkoxides in various charge-states. Indeed, these studies have resulted in the formation of a library of oxygen-deficient vanadium oxide clusters, with both bridging methoxide- and ethoxide- ligands, [V_6_O_6_(OR)_12_L]^*n*^ (R = CH_3_, C_2_H_5_; L = CH_3_CN, 
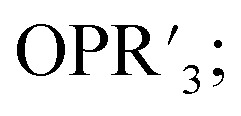
*n* = 1−, 0, 1+). Similar synthetic routes were also used to isolate a di-vacant cluster [V_6_O_5_(OCH_3_)_12_]^0^ (ox. state distrib.: V^III^_2_V^IV^_4_) which demonstrated the ability to post-synthetically control the metal-to-oxygen ratios within these polynuclear assemblies.^209^

**Fig. 21:**
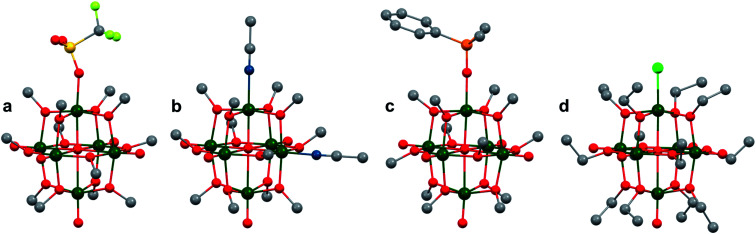
Molecular structures of complexes (a) [V_6_O_6_(OCH_3_)_12_]OTf, (b) [V_6_O_5_(OCH_3_)_12_(MeCN)_2_], and (c) [V_6_O_6_(OCH_3_)_12_(OP(CH_3_)_2_(C_6_H_5_))] and (d) [V_6_O_6_Cl(OC_2_H_5_)_12_]^1−^. Colour code: V: dark green, O: red, C: grey, N, blue: S: yellow, F: yellow-green. Hydrogen atoms, solvent molecules and counter cations have been omitted for clarity.

The molecular nature of these oxygen deficient cluster complexes allows for structural analysis of the metal–oxide assembly *via* single crystal X-ray diffraction. Bond metric analysis was possible for anionically doped clusters that crystallized with unique vanadium sites within the unit cell, including: [V_6_O_6_(OCH_3_)_12_OTf],^206^
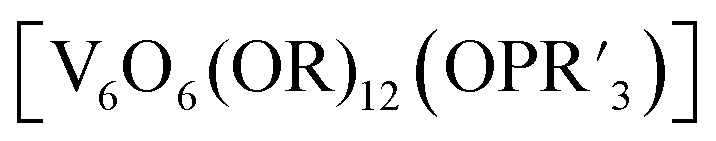
 (R = CH_3_, R′ = P(CH_3_)_3_, P(CH_3_)_2_(C_6_H_5_), P(CH_3_)(C_6_H_5_)_2_, P(C_6_H_5_)_3_; R = C_2_H_5_, R′ = P(CH_3_)_2_(C_6_H_5_))^207^ and V_6_O_5_(OCH_3_)_12_(CH_3_CN)_2_.^209^ (Fig. 21) Refinement of the crystallographic data revealed that V=O bond cleavage resulted in the formation of a site-differentiated vanadium ion(s) bearing a triflate, phosphine oxide, or acetonitrile ligand(s), respectively. In all cases, striking changes in the vanadium–oxygen bond lengths and angles were observed at the oxygen-deficient vanadium centre. In particular, comparison of the V–O_c_ bond length of the parent, fully oxygenated cluster to that of the vacant species, revealed that the site-differentiated vanadium ion(s) is pulled toward the centre of the cluster, as evident by the ∼0.2 Å decrease in bond length (oxygenated cluster (avg): 2.25 Å, oxygen-deficient clusters (avg): 2.08 Å). Notably, this bond truncation is similar to that observed upon introduction of cationic dopants into the Lindqvist core (*vide supra*). Additional evidence of this structural perturbation is observed in the analysis of the V^R^–O_b_–V_n_ bond angles (V^R^ = reduced vanadium ion; O_b_ = bridging methoxide oxygen atom; *n* = 2, 3, 4, 5), which are contracted by ∼4° in comparison to the fully-oxygenated Lindqvist clusters. Similar structural perturbations were observed in solvothermally-synthesized, oxygen-deficient POV–alkoxide cluster reported by Luneau and co-workers.^132^ Analysis of bond metrics of [V_6_O_6_(OCH_3_)_8_(calix)(CH_3_OH)]^1−^ revealed a similar V–O_c_ bond length (2.03 Å) and V^R^–O_b_–V_n_ angles (avg: 104.4°) to that reported in the oxygen-deficient clusters from the Matson laboratory.^206^

Evaluation of the structural perturbations of the oxygen-deficient POV–alkoxide clusters reported to date, shows a resemblance to predicted changes in V–O bond distances following vacancy formation on bulk V_2_O_5_. Theoretical studies on the reduced surface by Hermann and co-workers have shown that O-atom removal from the surface of V_2_O_5_ results in significant lattice distortions. For example, a new V–O–V linkage is proposed to form following O-atom removal due to contraction of the interlayer spacing between two V_2_O_5_ subunits.^14^ This proposed behaviour is mimicked by the movement of the apical, site-differentiated vanadium ion(s) in the structures of the Matson ([V_6_O_6−*x*_(OR)_12_L])^206,207,209^ and Luneau ([V_6_O_6_(OCH_3_)_8_(calix)(CH_3_OH)]^1−^)^132^ reduced metal oxide clusters.

Despite the similarities between polyoxometalates (POMs) and bulk metal oxides (*i.e.* composition of redox-active metal ions bridged by O^2−^, delocalized electronic structure), there is relatively little work investigating their use as homogenous analogues to bulk materials. This is rather surprising given the extensive literature showing that polyoxometalates can facilitate oxidative chemical transformations, similar to OAT reactions invoked with extended solids. Investigations into this oxidative reactivity of POMs have been extensively studied by Neumann^174,210–219^ and others,^220^ who have shown that POMs can mediate oxidative degradation of organic substrates under ambient reaction conditions. Mechanistic investigations have suggested that the mediation of these transformations with Keggin-type polyoxometalates, such as [PV_2_M_10_O_40_]^*n*^, likely undergoes a mechanism in which oxygen-atom vacancies are required to facilitate the OAT processes (*i.e.* following a Mars van Krevelen-type OAT mechanism). However, the active intermediates that are posed to feature oxygen-deficient sites have not been isolated. Indeed, a literature search of ‘vacant polyoxometalates’ solely features Lacunary-type polyoxometalates, where entire [MO_*x*_] subunits, rather than single O^2−^ atoms, are removed from the parent cluster.^97,221^ As such, the isolation of these atomically precise models of O-atom vacancies on metal oxide surfaces has afforded the Matson laboratory the ability to continue investigating the role of defect sites in heterogenous substrate activation. For a complete overview of this work, see a recent review published by Petel & Matson.^222^

### Atomically precise models of halide dopants in RMOs

With an overarching goal of using vanadium oxides for catalytic and thermochromic applications, researchers have been investigating alternative anionic dopants into V_*x*_O_*y*_.^223^ Preliminary investigations have shown that anionic, halide dopants (*e.g.* chlorine,^198,224^ fluorine^195–197,225^) on VO_2_ results in significant modifications of the electronic structure of the material. For example, stabilization of lattice phases, alteration of the band gap, enhancement of the visible transmittance, and modification of the phase transition temperature (MIT), have all been observed upon Cl- or F-doping on VO_2_. These results are notable given that halide-impurities are often observed in vanadium oxide systems due to the use of metal halide salts as single-source precursors for the synthesis of extended solids.^226–228^ Thus, to gain insight into the electronic influence of anionic dopants on bulk vanadium oxide, the consequence of introducing non-metal, non-oxygen defects on the surface of polyoxovanadium clusters will be summarized. Notably, while sulfide- and nitride-doped vanadium oxide solids have been synthesized and characterized, no reports, to date, feature the analogous S- or N-functionalized POVs; as such, this section will focus on a summary of halide-installed POVs.

**Fig. 22:**
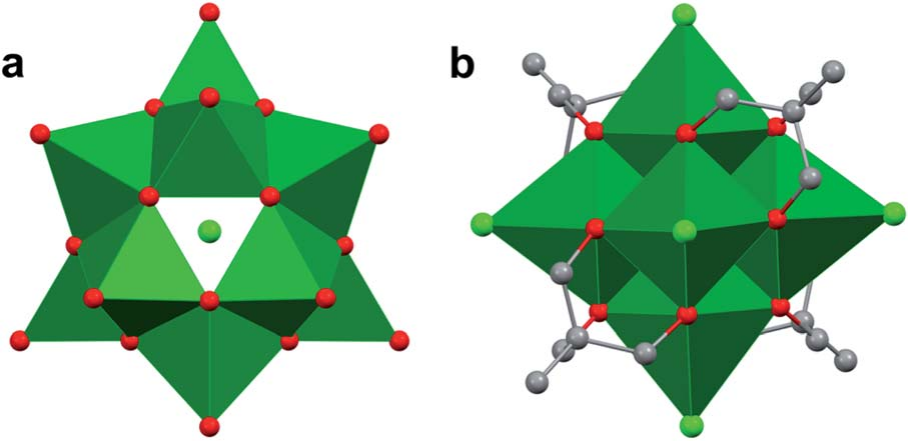
Representative molecular structures of POV cluster templated by (a) a chloride ion, [V_15_O_36_Cl]^6−^, and (b) terminal chlorides, [V_6_Cl_6_O(TRIS^CH_3_^)_4_]^2−^. Colour code: V: dark green polyhedral, O: red spheres, C: grey spheres, Cl: green spheres. Hydrogen atoms, solvent molecules and counter cations are omitted for clarity.

A special class of POMs includes ‘host–guest’ templates where high-nuclearity POMs form cage- or ring-like ‘host’ structures that can trap anionic guest species within their central cavities (Fig. 22a). The first reported examples of vanadium-based host–guest systems were reported in 1987 by Müller and co-workers who synthesized and characterized a chloride-functionalized POV, [V_15_O_36_Cl]^6−^ which featured a cyclic vanadium oxide structure (‘host’; [V_15_O_36_]) with a hollow centre that encompassed a chloride ion as an electrostatic ‘guest’.^229^ Since this initial investigation, many other halide-templated POV clusters have been reported including [V_18_O_42_(X)]^*n*^ (Müller, Khan; X = Cl, Br, I),^230,231^ [V_6_O_6_(OR)_12_Cl]^*n*^ (Matson),^91,92^ and [MV_12_O_32_X]^*n*^ (Cronin, Streb, Hayashi).^173,176,232,233^ While these types of clusters seem to qualify as ‘halide-doped POVs’, analysis of the bond metrics obtained *via* single crystal X-ray crystallography reveals that, in all cases, there is no direct bonding interactions between the vanadium–oxo host and the central chloride ion (V–Cl (avg) ∼3.4 Å). Instead, the role of the halide has been cited as an anionic template required for the assembly of the vanadyl cage.^176,234^ An exception to the halide-templated complexes described above is [V_12_O_32_(Br_2_)]^4−^, which was reported by Hayashi in 2020.^50^ The bowl-shaped POV was previously found to facilitate host–guest interactions; solvent bound to the electron-deficient cavity was easily displaced by heat and reduced pressure.^49^ Exposure of the guest-free POV to Br_2_ gas results in adsorption and polarization of Br_2_ in the structure, as seen for the first time by IR spectroscopy.^50^ This in turn enabled the molecule to perform bromination reactions on toluene, *n*-propane, *n*-butane, *n*-pentane, as well as 2- and 3-bromopentane, thus unlocking new reactivity for POVs. While fascinating work, the aforementioned host–guest halide–POV assemblies are not ideal molecular models for that of anionically doped metal oxides, where doping is defined as the substitution of a lattice oxygen-atom by halides, nitrides, or sulfides.

To aptly model a halide defect within vanadium oxides, a terminal oxo moiety must be replaced by a halide ion either during the self-assembly process or *via* post-synthetic modifications (*i.e.* V=O bond cleavage). McInnes and co-workers reported the first example of a solvothermally-synthesized, halide-doped hexavanadium cluster [V_6_Cl_6_O(TRIS^R^)_4_]^2−^; (R = CH_3_, C_2_H_5_), which is composed of six cluster-bound halide ligands (Fig. 22b).^235^ The oxidation state distribution of the vanadium centres within the solvothermally assembled cluster reveals that all the metal ions are in the 3+ oxidation state. These results confirm that anionically doping metal oxide cluster complexes with X-type ligands can significantly influence the oxidation state of metal ions within the assembly.

Comparison of structural parameters of [V_6_Cl_6_O(TRIS^R^)_4_]^2−^ to its congener bearing terminal oxido ligands, [V_6_O_7_(TRIS^R^)_4_]^2−^ reveals striking changes in the bond metrics of the Lindqvist core. In the case of the ‘non-doped’ system, [V_6_O_7_(TRIS^R^)_4_]^2−^, refinement revealed that the V^IV^–O_c_ bond lengths are 2.315(2) Å.^86,111^ In contrast, the hexa-chlorinated cluster, [V_6_Cl_6_O(TRIS^R^)_4_]^2−^, possesses an average V^III^–O_c_ bond length of 2.19 Å.^236^ The contraction of the V–O_c_ bond lengths upon halogenation (and reduction) suggest that halide doping of metal oxide nanostructures might impart significant structural changes in the lattice.

In addition to solvothermal methods for introducing chloride ions into POV clusters, the Matson laboratory discovered that post-synthetic modifications could result in the formation of halide-doped POV–alkoxide clusters. The authors discovered that addition of a transition metal halide salt, AlCl_3_, to [V_6_O_7_(OC_2_H_5_)_12_]^2−^ resulted in V=O bond cleavage, and subsequent V–Cl bond formation, to yield a new halide-doped POV–alkoxide cluster, [V_6_O_6_(OC_2_H_5_)_12_Cl]^1−^.^237^ Crystallographic analysis of the resulting cluster unambiguously confirmed that halide addition resulted in terminal oxo substitution and introduction of a cluster-bound chloride ion (Fig. 20, Table 6).

**Table 6:** Structural parameters of [V_6_O_7_(OCH_3_)_12_]^1−^, [V_6_O_6_(OCH_3_)_12_]OTf, [V_6_O_5_(OCH_3_)_12_]^0^, [V_6_O_6_(OCH_3_)_12_(OP(CH_3_)_2_(C_6_H_5_)]^0^ and [V_6_O_6_Cl(OC_2_H_5_))_12_]^1−^

Bond	Compound				
	[V_6_O_7_(OCH_3_)_12_]^1−^	[V_6_O_6_(OCH_3_)_12_]OTf (E = O)	V_6_O_5_(OCH_3_)_12_(CH_3_CN)_2_ (E = N)	V_6_O_6_(OCH_3_)_12_(OP(CH_3_)_2_(C_6_H_5_)) (E = O)	[V_6_O_6_Cl(OC_2_H_5_)_12_]^1−^ (E = Cl)
V1–E	—	2.052(8) Å	2.112(2) Å, 2.104(3) Å	2.0403(19) Å	2.2808(19) Å
V1–O_c_	—	2.079(4) Å	2.0666(17) Å, 2.0760(17) Å	2.0982(17) Å	2.2540(4) Å
O1–P1	—	—	—	1.5032(19) Å	—
V1–O_b_–V_n_	—	105°	105°	105.3°	108.3°
V1–O_b_–V1	—	—	96.9°	—	—
V_n_–O_c_ (avg.)	2.25 Å	2.32 Å	2.37 Å	2.32 Å	2.306 Å
V_n_–O_t_ (avg.)	1.60 Å	1.59 Å	1.61 Å	1.60 Å	1.602 Å
V_n_–O_b_–V_n_ (avg.)	110°	—	—	—	—

**Fig. 23:**
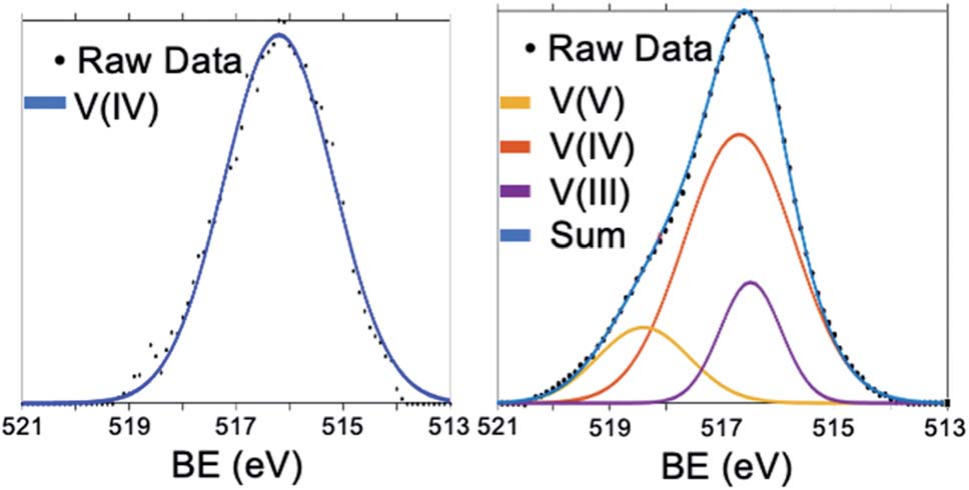
X-ray photoelectron spectra of the V 2p region of [V_6_O_7_(OC_2_H_5_)_12_]^2−^ (left) and [V_6_O_6_Cl(OC_2_H_5_)_12_]^1−^ (right). This figure has been adapted/reproduced from ref. 237 with permission from the American Chemical Society, copyright 2020.

To elucidate the electronic consequence of incorporating a halide into the POV–alkoxide assembly, infrared, electronic absorption, and X-ray photoelectron absorption (XPS) spectroscopies were employed. An oxidation state distribution of [V^III^V^IV^_4_V^V^] was garnered from XPS (Fig. 23). This spectroscopic analysis confirmed that V=O bond cleavage and V–Cl bond formation results in disproportionation of two V^IV^ ions within the initial [V^IV^_6_]^2−^ oxidation state distribution (Gaussian analysis of the V 2p_3/2_ region; V^III^ 16.8%, V^IV^ 67.3%, V^III^ 15.8%; [V^III^V^IV^_4_V^V^]).

Electronic changes, predicted by DFT calculations, of halide-doped VO_2_ showed that chlorine introduction results in an overall increased electrical conductivity, with an increased energy of the valence band resulting in a narrowing of the band gap (*i.e.* VO_2_:0.78 eV, Cl–VO_2_: 0.51 eV).^224^ While comparison of the electronic changes of metal oxides and polyoxometalates is challenging, due to the difference in energy states from a continuum of states *vs.* discreet frontier orbitals, respectively, electrochemical analysis of anionically-doped POV–alkoxides by cyclic voltammetry presents the ability to roughly compare the electronic consequences of halide doping between the metal oxide clusters and bulk material.

**Fig. 24:**
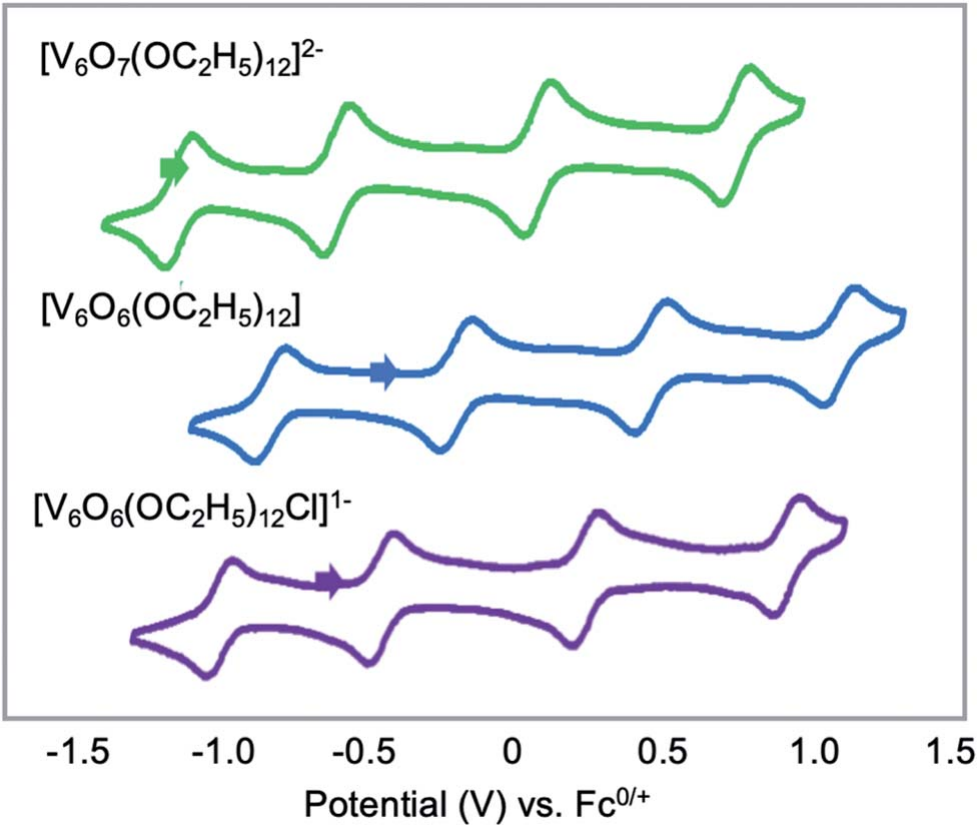
Cyclic voltammograms of [V_6_O_7_(OC_2_H_5_)_12_]^2−^ (green, top), [V_6_O_6_(OC_2_H_5_)_12_]^0^ (middle, blue), and [V_6_O_6_Cl(OC_2_H_5_)_12_]^1−^ (bottom, purple) collected in dichloromethane with 0.1 M [^*n*^Bu_4_N]PF_6_ as supporting electrolyte. This figure has been adapted/reproduced from ref. 237 with permission from the American Chemical Society, copyright 2020.

Electrochemical characterization of the halogenated and oxygen-deficient POV–alkoxide clusters revealed significant differences in the consequences of dopant identity (Fig. 24).^237^ Introduction of a surface defect, in the form of an oxygen-atom vacancy, resulted in a anodic shift of the redox events by ∼0.37 V in comparison to that of the parent cluster, [V_6_O_7_(OC_2_H_5_)_12_]^*n*^. Similar anodic shifts are observed upon chlorination, where, in this case, each redox event is shifted anodically by ∼0.2 V, relative to that of the parent, fully-oxygenated cluster. These relative shifts are consistent with the increased electron density introduced to the delocalized, hexavanadate core upon addition of the anionic substituent.

Collectively, the reports summarized herein encompass unique, unprecedented examples of self-assembled or post-synthetically modified polyoxovanadate clusters that feature anionic defects. These results show that accessing routes toward generating oxygen-atom vacant or halogen-doped POV–alkoxides allows for the use of these Lindqvist assemblies as molecular models for anionically-doped vanadium oxide materials. Indeed, the formation of these defected POVs present a significant advancement toward understanding the structural and electronic effects of introducing O-atom vacancies or chloride introduction to the surface of RMOs.

## Conclusion

This review has surveyed investigations targeting atomistic insights into the structural and electronic properties of bulk vanadium-oxides using POVs as homogeneous molecular models. We have discussed the synthetic methodologies and the preliminary understanding of formation mechanisms for the generation of POVs with targeted structural modifications which have emerged as a means to modify the physicochemical properties of the metal oxide core. We have also shown how alkoxylation of the V–O framework imparts structural distortions, enables redox flexibility through charge compensation, improves solubility in organic media, and influences charge transfer under electrochemical conditions. Similarly, we have described the formation of POVs with cationic and anionic dopants, presenting a range of complexes which serve to mimic heteroatom-substituted and oxygen-atom vacant extended solids. Collectively, these studies have cultivated important understanding into the structural and electronic consequences of manipulating POV cores.

The above-described strategies for modifying POVs present opportunities to better understand the surface chemistry and properties of extended solids and nanocrystalline materials; it is thus our opinion that the continuation of this work is important. However, the outlook of POV chemistry is not limited to studies of atomic precision in materials science; indeed these cluster complexes have demonstrated themselves as valuable in a variety of fundamental and applications-based research.^53,98,101,102,169^ Further advances in POV chemistry will seek to better understand how the properties of the metal oxide core can be manipulated toward a particular purpose. For example, understanding the precise mechanisms by which different POVs are formed is a critical step towards the purposeful generation of targeted core structures with desired features. In addition, the ability of researchers to access POVs with varying degrees of alkoxylation within a single V–O core structure is of fundamental interest seeing as no explicit investigation comparing the physicochemical properties of clusters with varying ligand densities has been reported to date. Moreover, where an array of studies have investigated O-atom vacant sites on POVs as a means of modelling material surfaces, these molecular systems are themselves intriguing potential candidates for expanding POM-based catalysis and O-atom transfer reactivity.^206,207,238^

One major area of emergent research of relevance to the development of POV assemblies focuses on the translation of knowledge of the properties and reactivity of molecular species to atomically precise multidimensional frameworks/materials. Two general classes of POV materials have been found: fully inorganic and inorganic–organic hybrid systems. The former is comprised of POVs linked *via* coordination of metal ions through terminal oxo groups.^239^ Hybrid systems can be formed by joining POVs by organic linkers^104,105,107^ or through coordination of transition metals to organic moieties of alkoxylated POVs.^240–242^ POV-incorporated frameworks represent an exciting frontier for the development of materials with tailorable, predictable properties with a broad range of applications. Notably, this research thrust greatly benefits from a foundational understanding of the molecular structure and properties of POVs, allowing for the identification of novel materials that may present exciting solutions in energy storage and catalysis.

## Conflicts of interest

There are no conflicts to declare.

## Supplementary Material
